# Hyperosmotic stimuli activate polycystin proteins to aid in urine concentration

**DOI:** 10.1172/jci.insight.186290

**Published:** 2025-08-05

**Authors:** Karla M. Márquez-Nogueras, Ryne M. Knutila, Virdjinija Vuchkovska, Charlie Yang, Patricia Outeda, Darren P. Wallace, Ivana Y. Kuo

**Affiliations:** 1 Department of Cell and Molecular Physiology, Stritch School of Medicine, Loyola University Chicago, Maywood, Illinois, USA.; 2 Graduate School, Loyola University Chicago, Illinois, USA.; 3 Center for Proteomics and Molecular Therapeutics, Chicago Medical School, Rosalind Franklin University of Medicine and Science, Chicago, Illinois, USA.; 4 Department of Medicine, University of Maryland School of Medicine, Baltimore, Maryland, USA.; 5 Department of Internal Medicine, University of Kansas Medical Center, Kansas City, Kansas, USA.

**Keywords:** Cell biology, Nephrology, Calcium signaling, Chronic kidney disease, Epithelial transport of ions and water

## Abstract

Autosomal dominant polycystic kidney disease (ADPKD) is caused by mutations in *PKD1* or *PKD2*, which encode polycystin-1 (PC1) and polycystin-2 (PC2), respectively. These proteins are thought to form a signaling complex that can flux cations, including calcium. One of the earliest symptoms in ADPKD is a decline in the concentrating ability of the kidneys, occurring prior to cyst formation. We reasoned that hyperosmolality stimulates the polycystin complex, and that the loss of this function impairs water reabsorption. We found that hyperosmolality resulted in the phosphorylation of microtubule-associated protein 4 (MAP4) in a PC1-dependent manner, which then elicited ER-localized PC2 calcium signals. ER-localized PC2 hyperosmotic calcium signals were required for trafficking of the water channel aquaporin (AQP2). Precystic PC1-KO and PC2-KO murine kidneys had cytosol-localized AQP2 and diluted urine compared with their respective controls. Kidney tissue sections from ADPKD patients showed decreased AQP2 apical membrane localization in cystic and noncystic tubules. Our study demonstrates that osmolality is a physiological stimulus of the polycystin complex, and loss of polycystin osmosensing results in impaired water reabsorption via AQP2. This likely contributes to the declined concentrating ability of the kidneys and high circulating vasopressin levels in patients with ADPKD.

## Abstract 2

Kidneys enable urine to be concentrated. We show that the polycystin proteins, implicated in a genetic kidney disease, is essential in this pathway.

## Graphical abstract

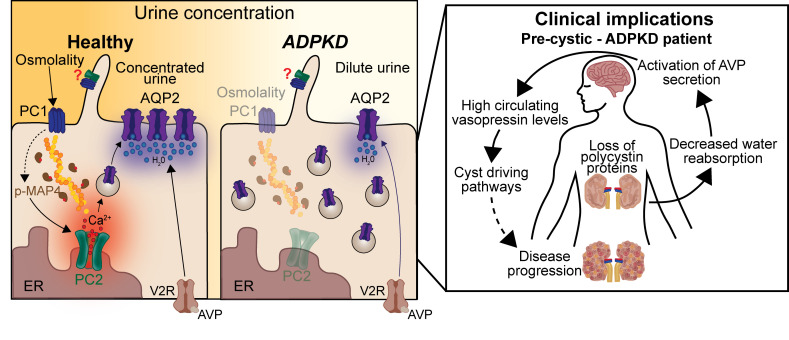

## Introduction

Autosomal dominant polycystic kidney disease (ADPKD) is the leading genetic cause of kidney failure and has no cure (1). Over 95% of all ADPKD cases are due to mutations in the *PKD1* and *PKD2* genes, encoding polycystin-1 (PC1) and -2 (PC2), respectively (2). An early symptom in patients with ADPKD that happens decades prior to a decline in renal function is a decrease in urinary concentration (3, 4). Paradoxically, patients with ADPKD also present with high circulating levels of arginine vasopressin (AVP; and its more stable surrogate, copeptin) (5–8), which binds to the vasopressin type 2 receptors (V2Rs) to increase water reabsorption in the collecting duct (CD) through the apical incorporation of aquaporin 2 (AQP2). Currently, the cause of the renal concentrating deficit in patients with ADPKD remains unknown.

Calcium signaling regulates key cellular processes. In ADPKD, many of the cystic pathways (cell proliferation, apoptosis, and autophagy) can be linked to dysregulation of calcium signaling (2). Hyperosmolality, as experienced in medullary CD cells, can induce the trafficking of AQP2 to the membrane via a non–vasopressin-dependent pathway (9–11). It can also lead to rises in cytosolic calcium levels, but the molecules that allow for this calcium signal has not been clearly identified (12). PC1 in complex with PC2 can form a 1:3 heterotetrameric cation channel, but PC2 by itself also forms a cation-permeant channel (2). The PC1-PC2 complex localizes to the primary cilia; however, PC2 prominently resides on the endoplasmic reticulum (ER) (13) and can either directly or indirectly with other calcium channels induce calcium release from the ER (14–17). There is no consensus as to what physiological cues activate calcium signaling by PC2.

We tested whether osmolality acts as a physiological stimulus in eliciting polycystin-dependent calcium signals required for the osmotic trafficking of AQP2. We identified that hyperosmolality leads to a PC1-dependent phosphorylation of microtubule-associated protein 4 (MAP4), which interacts with ER-localized PC2 to induce an intracellular calcium signal. In precystic animal models, loss of PC1, MAP4, or PC2 resulted in cytosolic AQP2 localization and urine concentrating defects. This study demonstrates that hyperosomolality is a physiologically relevant stimulus of the polycystin proteins that aids in AQP2 trafficking. Loss of this osmosensing pathway may explain the paradox of the urinary concentration deficit and subsequent elevation of vasopressin seen in patients with ADPKD.

## Results

### Precystic PC1-KO and PC2-KO mice have decreased urine osmolality and PC2-KO mice have no calcium response to hyperosmotic stimuli.

**Figure 1:**
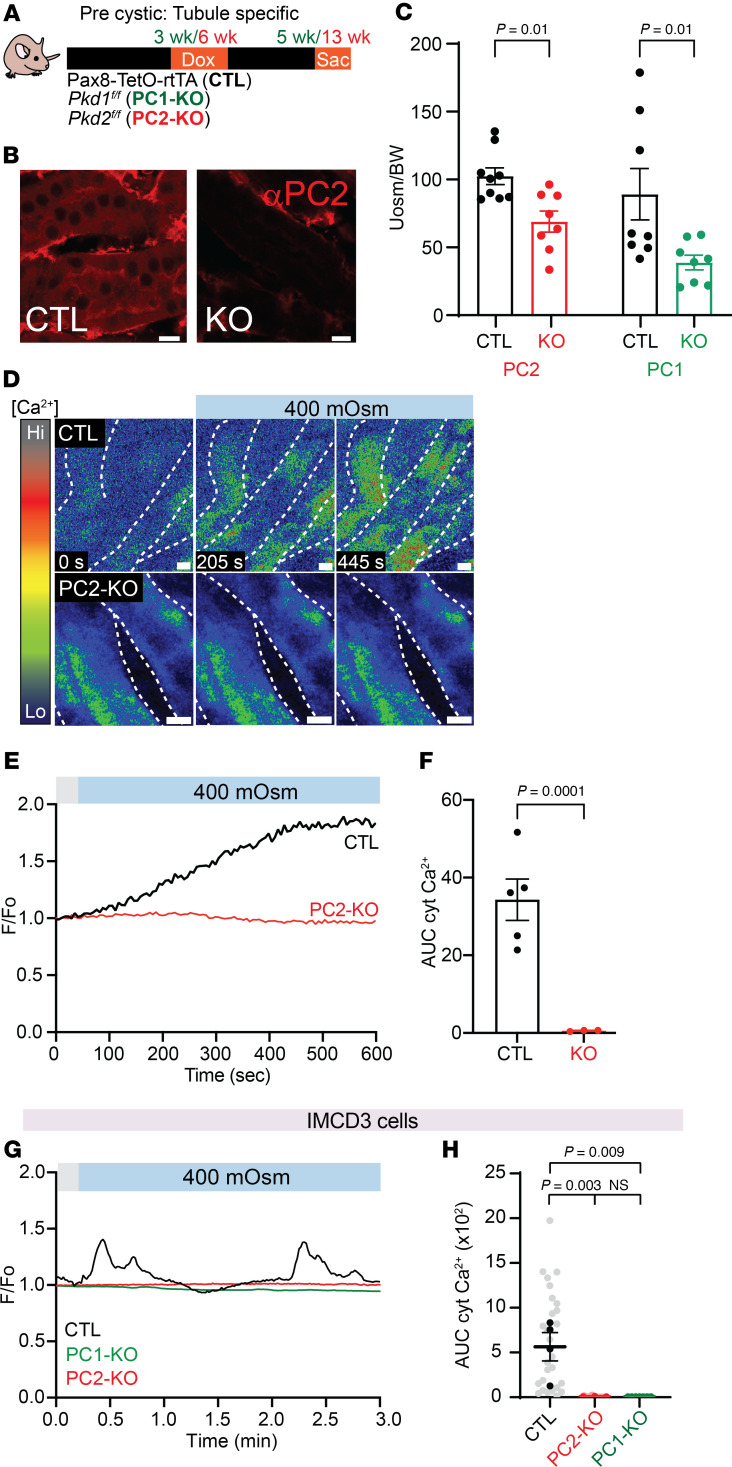
Precystic PC1 and PC2-KO mice have decreased urine concentration and decreased calcium signaling upon hyperosmotic challenge. (**A**) Model of precystic tubule–specific deletion of PC1 and PC2 by crossing Pax8-TetO-rtTA (CTL) mice with *Pkd1^fl/fl^* mice (PC1-KO) or *Pkd2^fl/fl^* mice (PC2-KO). (**B**) Representative images with decreased expression of PC2 in PC2-KO mouse. Residual PC2 staining in the PC2-KO mouse arises from non-epithelial cells. Scale bars: 10 μm. (**C**) Precystic PC2 (red) and PC1-KO (green*)* mice had decreased urine osmolality compared with CTL mice. Each dot represents an individual mouse. *n* = 8–9. Data analyzed by Mann-Whitney *U* test. (**D**) Representative time-lapse images of collecting duct (CD) tubules expressing gCaMP from kidney slices of CTL mice (top) and PC2-KO mice (bottom). Extracellular osmolality was increased from 250 to 400 mOsm with NaCl. Scale bars: 20 μm. (**E**) Representative trace of cytosolic calcium changes in CTL CD tubules (black line) and PC2-KO tubules (red line). (**F**) Quantification of area under the curve (AUC) of calcium transients comparing CTL to PC2-KO CD tubules; each dot is 1 mouse. *n* = 4–5. Data analyzed by Mann-Whitney *U* test. (**G**) Representative trace of cytosolic calcium increases in CTL IMCD3 cells (black line) with hyperosmotic stimuli not seen in PC1-KO IMCD3 cells (green line) and PC2-KO IMCD3 cells (red line). (**H**) AUC decreased in PC1-KO and PC2-KO IMCD3 cells. Data were analyzed to determine normality. Bar graphs represent mean ± SEM. Dark dots represent biological replicates, while light dots represent individual cells *n* = 30. *P* values listed in each panel.

Patients with ADPKD have decreased ability to concentrate urine (4, 6). However, while cysts can be observed in utero (18, 19), it is unclear whether this defect is due to the presence of renal cysts and disruption of the kidney architecture, or if the polycystin proteins themselves contribute to a signaling pathway involved in urine concentration (3). To test between these 2 options, we used precystic mouse models (20–22) by knocking out either PC1 or PC2 in renal epithelial cells in an inducible adult mouse model (Pax8-TetO-rtTA mice crossed with *Pkd1^fl/fl^* or *Pkd2^fl/fl^* mice). Doxycycline-induced Pax8-TetO-rtTA-Cre mice (CTL), Pax8-TetO-rtTA-Cre-*Pkd1^fl/fl^* (PC1-KO), and Pax8-TetO-rtTA-Cre-*Pkd2^fl/fl^* mice (PC2-KO) were used at either 6–8 weeks after doxycycline (*Pkd2*) or 2 weeks after doxycycline (*Pkd1*) (Figure 1A). Immunofluorescence analysis confirmed loss of PC2 expression in PC2-KO mice and that it was confined to renal epithelial cells (Figure 1B) and no cysts (Supplemental Figure 1A; supplemental material available online with this article; https://doi.org/10.1172/jci.insight.186290DS1). We analyzed urine osmolality with ad libitum water access and found that PC1-KO and PC2-KO mice had significantly reduced urine osmolality compared with CTL mice (Figure 1C). No changes in body mass composition and water and food consumption were observed between the CTL and PC2-KO mice, suggesting that the decreased urine osmolality was not due to these factors (Supplemental Figure 1, B–E).

### Renal tubules from PC2-KO mice have no calcium response to hyperosmotic stimuli.

As both PC1-KO and PC2-KO mice produce a more dilute urine, we hypothesized that the polycystin proteins are involved in the osmosensing pathway in CD cells. Previous work has shown that transient receptor potential (TRP) channels can mediate cytosolic calcium increases induced by hyperosmolality in the CD (23, 24). To test whether PC2, a known calcium channel, is mediating this response, we crossed CTL and PC2-KO mice with Salsa6f mice (25). Salsa6f mice express gCaMP6F and tdTomato under a *cre* promoter, allowing ratiometric cytosolic calcium measurements (25) in renal epithelial cells. In kidney slices of Salsa6f-CTL mice, we found that switching from a hypoosmotic to hyperosmotic solution (250 to 400 mOsm, increased by NaCl) stimulated an increase in cytosolic calcium in tubules, but not in the tubules of Salsa6f-PC2-KO mice (Figure 1, D–F, and Supplemental Video 1). Exogenous application of AVP, which activates V2R, induced similar calcium responses in CTL and PC2-KO mice (Supplemental Figure 1F). These data demonstrate that PC2-KO impairs the hyperosmotically induced calcium response in the kidney.

### PC2-KO abolished the hyperosmotic calcium response.

To confirm the role of PC2 in the osmosensing pathway, we examined the effect of PC2 in the hyperosmotically induced calcium response in primary CD cells, inner medullary collecting duct (IMCD3), human embryonic kidney (HEK) cells, and murine myoblast (C2C12) cells (26). Validation of the PC2-KO cell lines and PC2’s ER location was performed via Western blot, quantitative PCR (qPCR), or by immunofluorescence analysis (Supplemental Figure 2, A–D). Hyperosmotic stimuli (300 to 400 mOsm) caused a robust increase in cytosolic calcium in primary CD CTL cells, but not in the PC2-KO (*Pkd2* CRISPR) cells (Supplemental Figure 3, A–D), like the response in the PC2-KO kidney slices (Figure 1, D–F). This response was also observed in C2C12 and IMCD3 cells (27) (Supplemental Figure 3, E–G, Figure 1, G and H, and Supplemental Video 2). To demonstrate that the cytosolic calcium increase was due to osmotic pressure and not the salt gradient, the extracellular osmotic concentration was increased with mannitol, and a calcium increase was still observed (Supplemental Figure 4, A and B). Since PC2 can form a complex with PC1, we tested whether deletion of PC1 also disrupted the hyperosmotically induced calcium response. We generated IMCD3 PC1-KO cells, which were validated by qPCR and immunofluorescence assay (Supplemental Figure 2, E and F). Hyperosmotic stimuli also abolished the calcium response in IMCD3 PC1-KO cells (Figure 1, F and G). These results show that the hyperosmotically induced calcium increase is dependent on PC1 and PC2.

### Primary cilia and plasma membrane calcium channels do not contribute to the PC2-driven osmotic response.

**Figure 2:**
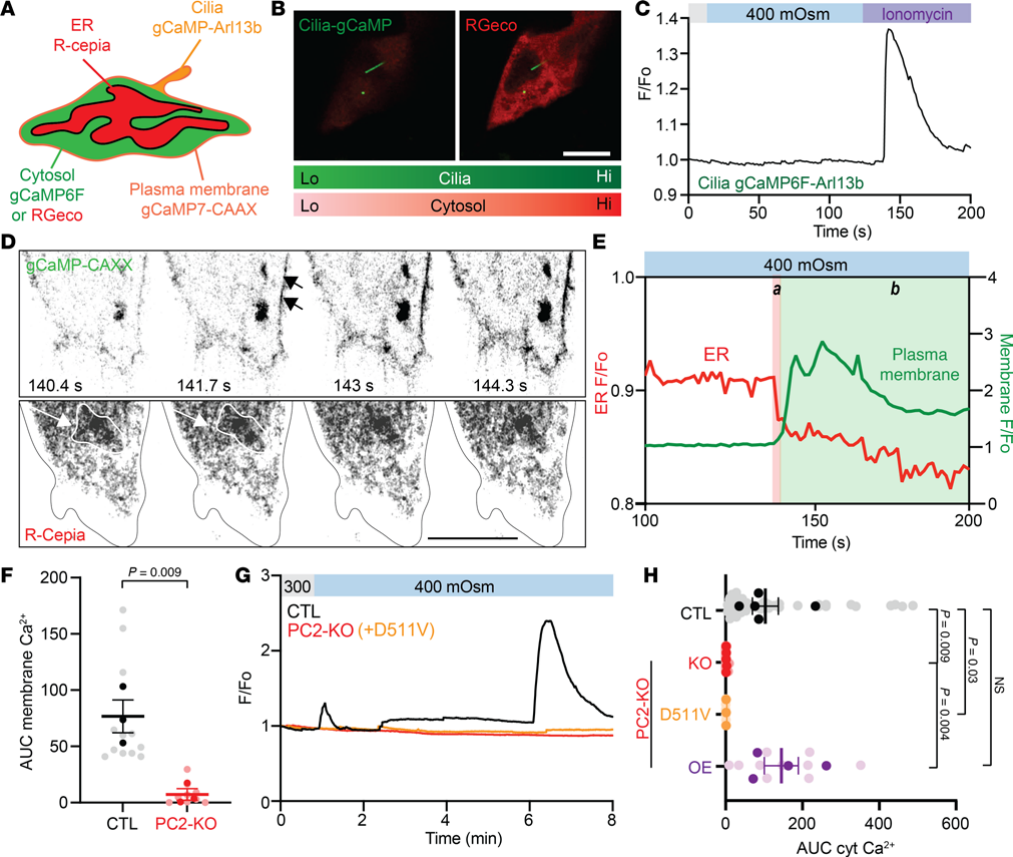
Hyperosmotic stimuli induce ER-localized PC2 calcium release. (**A**) Diagram of the different genetic calcium indicators used for calcium measurements. (**B**) Representative images of cytosolic calcium (red) increase but not ciliary calcium (green) in IMCD3 CTL cells. Scale bar: 10 μm. (**C**) Ciliary calcium did not increase in CTL C2C12 cells after stimulation with 400 mOsm but did increase with ionomycin (1 μM), representative of *n* = 6. (**D**) Representative images of C2C12 CTL cells expressing the plasma membrane calcium indicator (gCaMP7s-CAAX, top panels) and ER calcium indicator (R-CEPIA, bottom panels). Scale bar: 10 μm. (**E**) Representative traces of simultaneous calcium changes in R-CEPIA (red line, region a) and plasma membrane calcium (green line, region b) in C2C12 CTL cells after hyperosmotic stimulus. White region on the left indicates ER calcium drops after hyperosmotic stimuli. Green region on the right indicates plasma membrane calcium spark. (**F**) Membrane calcium AUC decreased in PC2-KO cells. *n* = 12. Statistical analysis by Mann-Whitney *U* test. Dark dots represent biological replicates, while light dots represent individual cells. (**G**) Representative trace of cytosolic calcium changes in CTL (black line), PC2-KO (red line), and PC2-KO+D511V-PC2 variant (orange line) C2C12 cells. (**H**) Reexpression of full-length PC2 (OE), but not D511V variant, restored the cytosolic calcium response in the C2C12 PC2-KO cells. *n* = 10–30. Statistical analysis determined by a Kruskal-Wallis test followed by Dunn’s test. Bar graphs represent mean ± SEM. Dark dots represent biological replicates, while light dots represent individual cells. *P* values listed in each panel.

We then analyzed different cellular compartments (cilia, plasma membrane, or ER) to identify where the osmotically induced calcium signal was originating from using various genetically encoded calcium indicators (Figure 2A). Previous work has demonstrated that the polycystin proteins participate in ciliary calcium responses (28-31). We used gCaMP6F-Arl13B (targeted to the primary cilia; Figure 2B) (32), and found that hyperosmotic stimuli did not induce a ciliary calcium response in CTL cells but addition of ionomycin (a calcium ionophore) did (Figure 2C; C2C12 cells). However, in 2 of 8 cilia tested in IMCD3 cells, we observed a back propagation of cytosolic calcium to the cilia (Supplemental Figure 5, A–C). The length of cilia in IMCD3 cells and percentage of cilia in C2C12 cells between the CTL and PC2-KO cells were not different (Supplemental Figure 5, D–F). Since the cilia were not involved in the osmotically driven calcium response, we focused on the ER and plasma membrane and continued our analysis in C2C12 cells because of the larger cell size relative to IMCD3 cells. Hyperosmolality induced a reduction in ER calcium (detected by R-CEPIA, which localizes to the ER) (33), indicating calcium release from the ER (Figure 2, D and E, region “a”). The response was subsequently followed by an increase in plasma membrane calcium (detected by gCaMP7s-CAAX, which is tethered to the plasma membrane; Figure 2, D and E, region “b”). This observation suggested that hyperosmotic stimuli first induced ER calcium release, which then stimulated plasma membrane calcium influx. Hyperosmotic stimuli in the absence of extracellular calcium still elicited a calcium response in CTL cells but not in PC2-KO cells (Supplemental Figure 4, C and D). This result further supports the idea that the calcium response after hyperosmolality is due to intracellular calcium release and not calcium influx. PC2-KO cells lacked plasma membrane calcium sparks after hyperosmotic stimuli, most likely due to the inability of PC2-KO cells to enable calcium-activated calcium influx (Figure 2F).

To test whether the ion-conducting activity of PC2 is required for the hyperosmotically induced calcium increase, we expressed the pathogenic variant D511V, a mutation in the voltage sensing domain, which inhibits the opening of the channel (34, 35). Reexpression of full-length PC2, but not the D511V variant, restored the calcium response in PC2-KO cells (Figure 2, G and H). These results suggest that hyperosmotic stimuli induce calcium release from ER-localized PC2 without the involvement of the cilia or plasma membrane.

### PC2 recruits the InsP_3_R to sustain the osmotically induced calcium response.

Previous studies have demonstrated that PC2 can recruit other ER-localized calcium channels like the inositol 1,4,5-trisphosphate receptor (InsP_3_R) (14–16). Therefore, to determine the role of the InsP_3_R in the osmotic response, we used a previously characterized cell line with the 3 isoforms of the InsP_3_R knocked out (3KO-HEK, generated from HEK cells) (36). We saw a 50% reduction of the calcium response in the 3KO-HEK cells compared with a complete loss of a response by the PC2-KO HEK cells after hyperosmotic stimuli (Supplemental Figure 6, A and B). These data suggest that PC2 can recruit the InsP_3_R to sustain the calcium response induced by hyperosmotic stimuli (Supplemental Figure 6C).

### PC2 interacts with and regulates microtubule protein MAP4.

**Figure 3:**
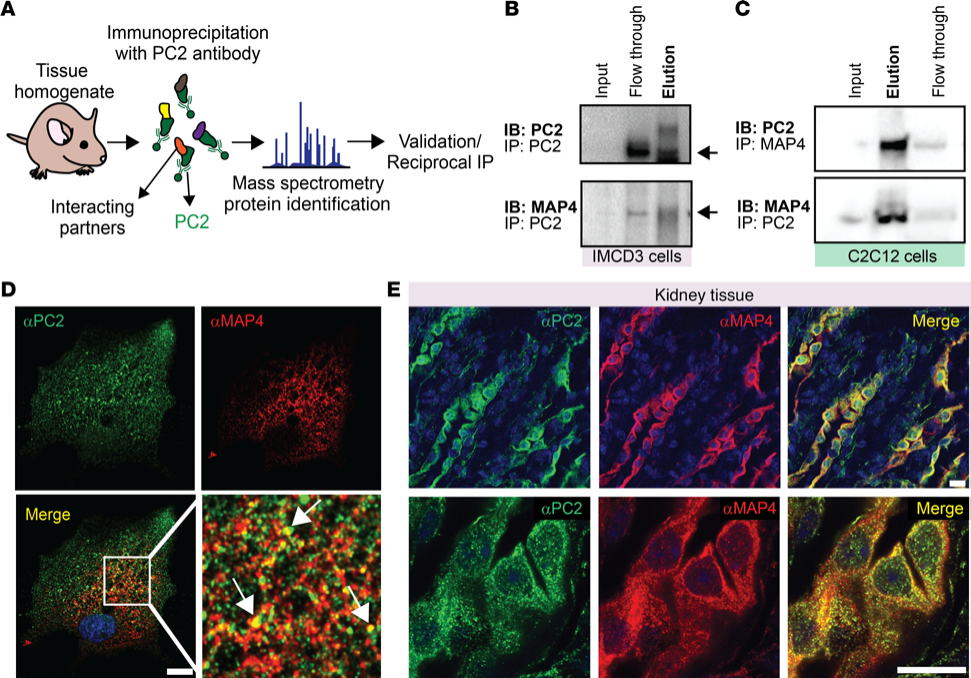
PC2 interacts with microtubule-associated protein 4 (MAP4). (**A**) Diagram of PC2 immunoprecipitation from murine tissue homogenate to identify potential interacting partners. (**B** and **C**) Immunoprecipitation assay validating interaction between PC2 and MAP4 in IMCD3 (**B**) and C2C12 (**C**) cells. Black arrows highlight MAP4 expression. (**D**) Immunofluorescent staining of PC2 (green) and MAP4 (red) in CTL C2C12 cells. White arrows highlight colocalization between the 2 proteins. Scale bars: 10 μm. Original magnification for zoomed-in images, ×113.4. (**E**) Immunofluorescent staining of PC2 (green) and MAP4 (red) in kidney tissue from mice.

Since PC2 localizes to the ER and the calcium signals originated from the ER, it is unlikely to be the osmotic sensor. To identify an immediate upstream interactor of PC2 in the osmotic pathway, we used a nonbiased approach and performed immunoprecipitation reactions followed by mass spectrometry (MS) with endogenous PC2 from mouse tissue (Supplemental Table 1 and Figure 3A). We focused on cytoskeletal or microtubule proteins since the cytoskeleton mediates rapid changes induced by osmotic stimuli (37), and link the cytoskeleton to the ER (38). We identified MAP4 as the potential PC2 interacting partner. We validated the interaction of these 2 proteins by performing reciprocal immunoprecipitation experiments of endogenous PC2 and MAP4 in both IMCD3 and C2C12 CTL cells (Figure 3, B and C). Immunofluorescence studies of endogenous PC2 and MAP4 demonstrated areas of colocalization in C2C12 cells and murine kidney sections (Figure 3, D and E).

### The ER-localized PC2-MAP4 interaction is osmotically driven.

**Figure 4:**
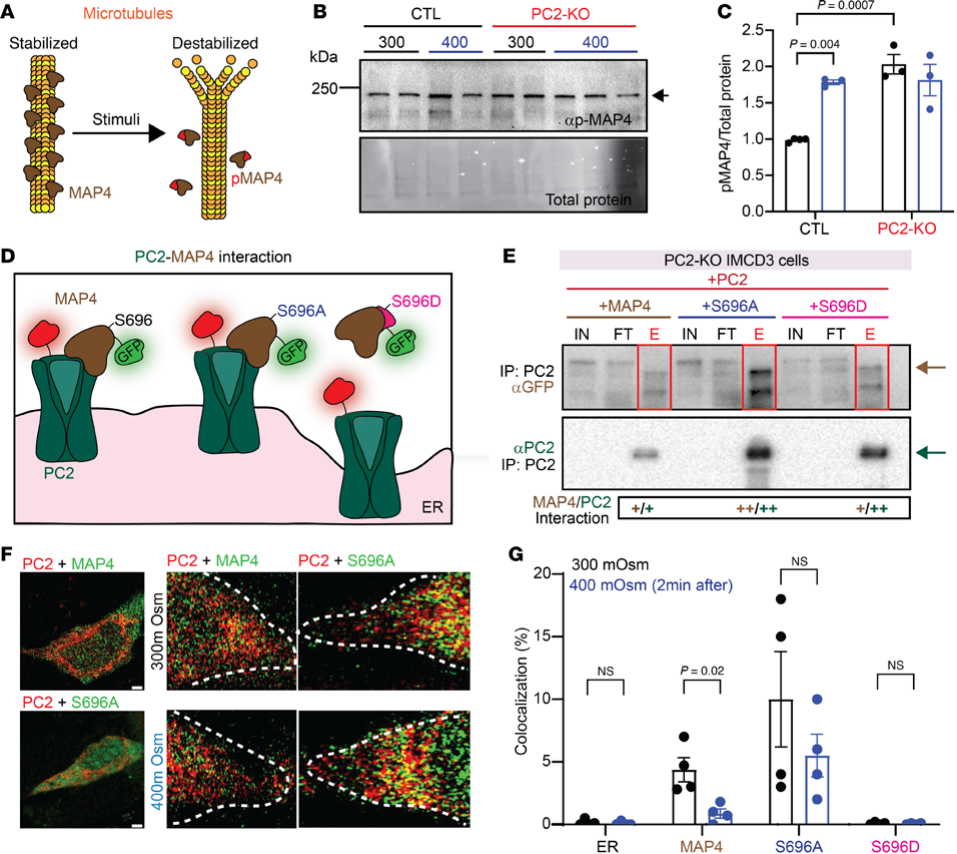
The PC2 and MAP4 interaction is dependent on MAP4 phosphorylation. (**A**) Function of MAP4 in microtubule stability. (**B**) Expression of p-MAP4 in C2C12 CTL and PC2-KO cells at 300 mOsm and 400 mOsm. Total protein was used as loading control. (**C**) p-MAP4 increased in C2C12 CTL cells after hyperosmotic stimuli. p-MAP4 levels in PC2-KO cells did not change with osmotic shifts. Similar results seen in IMCD3 cells (data not shown). Statistical analysis by Kruskal-Wallis test followed by Dunn’s test. (**D**) Diagram of PC2-MAP4 interaction with the phospho-null and phospho-mimetic mutants. (**E**) Immunoprecipitation assay from IMCD3 PC2-KO cells expressing PC2-mCherry and MAP4-eGFP, MAP4^S696A^-eGFP, or MAP4^S696D^-eGFP. PC2-MAP4 interaction was stabilized with the expression of MAP4^S696A^-eGFP. IN, input; FT, flow-through; E, elution. (**F**) Representative images of C2C12 PC2-KO cells cotransfected with MAP4-eGFP, PC2-mCherry (top left panel), or MAP4^S696A^-eGFP (bottom left panel). Colocalization between PC2-mCherry and MAP4-eGFP decreased after hyperosmotic stimuli (middle panels) but unchanged with MAP4^S696A^. Scale bars: 2 μm. (**G**) Quantification of ER-mCherry and MAP4-eGFP (ER), PC2-mCherry and MAP4-eGFP (MAP4), PC2-mCherry and MAP4^S696^A-eGFP (S696A), and PC2-mCherry and MAP4^S696^D-eGFP (S696D) at 300 mOsm (black bars) and after hyperosmotic stimuli (400 mOsm, blue bars). Statistical analysis was by Mann-Whitney *U* test. In **C** and **G**, each dot is an independent biological replicate (*n* = 3–4). Bar graphs represent mean ± SEM. *P* values listed in each panel.

MAP4 binds to microtubules to promote stabilization and upon phosphorylation, MAP4 disassociates from the microtubules, promoting depolymerization (39, 40) (Figure 4A). We first tested whether hyperosmolality induced phosphorylation of MAP4 (41–43). We observed a significant increase in p-MAP4 after hyperosmotic stimuli in CTL C2C12 cells (Figure 4, B and C). However, in PC2-KO cells, p-MAP4 was significantly increased under isosmotic conditions and remained unchanged after hyperosmotic stimuli in both cell lines (Figure 4, B and C). As the deletion of PC1 also abolished the osmotically induced calcium response, we tested whether PC1 sits upstream of the phosphorylation of MAP4. Unlike the PC2-KO cells, p-MAP4 in PC1-KO cells was significantly decreased in comparison with the CTL cells (Supplemental Figure 7, A and B). This suggests that the osmotically induced phosphorylation of MAP4 sits downstream of PC1 but upstream of PC2.

We also measured total MAP4 in PC2-KO cells, which was significantly less compared with CTL cells and unchanged with hyperosmotic stimuli in either genotype (Supplemental Figure 7, C and D). Reexpression of full-length PC2 and the D511V mutant restored MAP4 expression, suggesting that the expression of PC2, but not its calcium conduction, is required for MAP4 expression (Supplemental Figure 7E). Reexpression of full-length PC2 also restored the distribution of MAP4, and these 2 proteins colocalized with each other as assessed by Mander’s coefficient analysis (Supplemental Figure 8, A and B).

We then tested whether the interaction between PC2 and MAP4 is dependent on the phosphorylation status of MAP4. We fluorescently tagged MAP4 and generated a phosphomimetic mutant (MAP4^S696D^), as well as a phospho-null mutant (MAP4^S696A^) (Figure 4D). We validated the expression of MAP4-eGFP, MAP4^S696D^-eGFP, and MAP4^S696A^-eGFP in PC2-KO IMCD3 cells through Western blot (Supplemental Figure 8C). As expected, expression of p-MAP4 was significantly increased in the phospho-mimetic variant (S696D) in comparison with the WT MAP4 or the phospho-null mutant (Supplemental Figure 8C). By immunoprecipitation, we found that expression of the phospho-null mutant had the highest amount of interaction with PC2, while the interaction was decreased in the phospho-mimetic mutant (Figure 4E).

We then measured whether the dynamic interaction between MAP4 and PC2 is osmotically driven and dependent on the MAP4 phosphorylation status by using super-resolution imaging (Figure 4F). We observed that in PC2-KO IMCD3 cells there was minimal colocalization between MAP4-eGFP and mCherry-ER, which remained unchanged after hyperosmotic stimuli (Figure 4G, ER). In contrast, we observed an approximately 4%–5% colocalization between MAP4-eGFP and PC2-mCherry, which significantly decreased to less than 1% after approximately 2 minutes of hyperosmotic stimuli (Figure 4G, MAP4). The phospho-null mutant had a higher level of colocalization (~10%), which was not altered after hyperosmotic stimuli (Figure 4G, S696A). There was minimal colocalization between the phospho-mimetic variant and PC2-mCherry, which was unchanged after hyperosmotic stimuli (Figure 4G, S696D). Collectively, these data suggest that the dynamic interaction between MAP4 and PC2 is osmotically driven and dependent on the phosphorylation status of MAP4.

**Figure 5:**
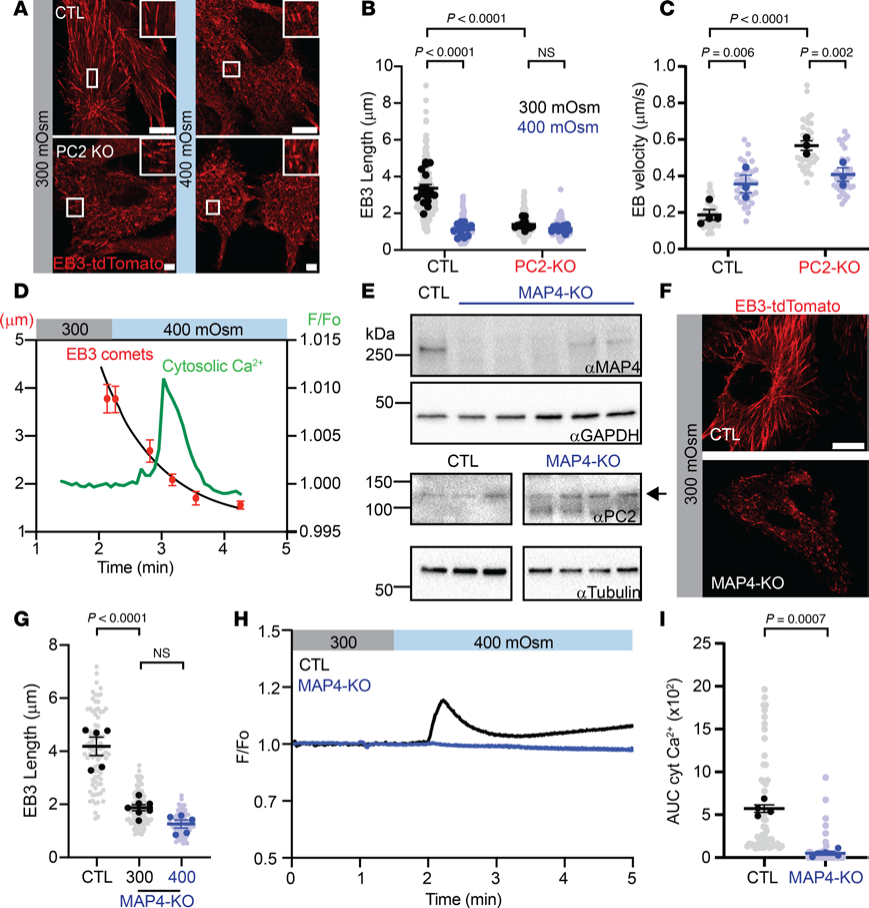
MAP4 is an upstream interactor of the PC2-mediated osmosensing pathway. (**A**) Representative images of EB3-tdTomato expression in C2C12 CTL cells (top panels) and PC2-KO cells (bottom panels) at 300 mOsm and 400 mOsm. Insets: Zoomed images of EB3 comets. Scale bars: 10 μm. Original magnification for zoomed-in images, ×113.4. (**B**) EB3 comet length decreased in C2C12 CTL with 400 mOsm (blue vs. black bars). At 300 mOsm (black bars), comets were reduced in PC2-KO cells and remained unchanged at 400 mOsm (blue bars). Data analyzed by Kruskal-Wallis test followed by Dunn’s test. (**C**) Mean velocity of EB3 comets increased with 400 mOsm in CTL C2C12 cells, while in PC2-KO cells comets were faster at 300 mOsm and decreased with 400 mOsm. Data analyzed to determine normality, then by 2-way ANOVA followed by Šídák’s test. (**D**) Shortening of EB3 comets in C2C12 CTL cells (red dots) occurs within 0.91 minutes after hyperosmotic stimuli, which induced a cytosolic calcium (green trace) increase 20 seconds after. (**E**) MAP4 expression in the KO cell line (top Western blot). PC2 expression remained unchanged in MAP4-KO cells (bottom Western blot). GAPDH and tubulin used as loading controls, respectively. (**F**) Representative images of EB3-tdTomato expression C2C12 CTL cells (top panel) and MAP4-KO cells (bottom panel). Scale bar: 10 μm. (**G**) EB3 comet length was decreased in C2C12 MAP4-KO at baseline and remained unchanged with 400 mOsm. Statistical analysis determined by Kruskal-Wallis test followed by Dunn’s test. (**H**) Representative trace of cytosolic calcium changes in C2C12 MAP4-KO cells after hyperosmotic stimuli (blue line). (**I**) AUC decreased in the MAP4-KO after hyperosmotic stimuli. Statistical analysis by Mann-Whitney *U* test. Bar graphs represent mean ± SEM. In panels **B**, **C**, **G**, and **I**, dark dots represent biological replicates, while light dots represent individual cells. *n* > 3 biological replicates. *P* values listed in each panel.

Hyperosmolality and deletion of PC2 led to increased levels of p-MAP4, which stimulates depolymerization of microtubules; therefore, we examined microtubule growth dynamics. We used EB3-tdTomato, which binds to the plus end of microtubules, and quantified both the length and mean velocity of the microtubules (Figure 5A and Supplemental Video 3). In CTL cells, the length of EB3 comets in isosmotic conditions was approximately 3 μm and significantly decreased to approximately 1.5 μm with hyperosmolality (Figure 5B and Supplemental Table 2). In PC2-KO cells, EB3 length under isosmotic conditions was shorter (~1.5 μm) and remained unchanged under hyperosmotic stimuli (Figure 5B). The velocity of the EB3 comets under hyperosmotic conditions in CTL cells increased, indicative of microtubule growth (Figure 5C). In contrast, the mean velocity of EB3 comets in PC2-KO cells before or after hyperosmotic stimuli was at least 2–3 times more than the CTL cells after hyperosmolality (Figure 5C). These results indicate that loss of PC2 leads to a state of depolymerized microtubules determined by shorter and faster EB3 comets and consistent with an increased level of p-MAP4.

Next, we assessed the temporal relationship between microtubule rearrangement and calcium signaling induced by hyperosmolality. After hyperosmolality, the length of the EB3 comets quickly decreased to approximately 1.5 μm, indicating microtubule collapse, followed by an increase in cytosolic calcium (Figure 5D). The absence of calcium signals from PC2-KO cells is likely due to impaired microtubules dynamics that fail to relay the external stimuli to intracellular signals like calcium release.

### MAP4-KO phenocopies PC2-KO.

To confirm that MAP4 is an upstream responder in the PC2-mediated osmosensing pathway, we generated MAP4-KO cells (Figure 5E and Supplemental Figure 9, A and B). Although PC2-KO led to decreased MAP4 expression, the localization and expression of endogenous PC2 remained unchanged in the MAP4-KO cells (Figure 5E and Supplemental Figure 9C). We examined whether deletion of MAP4 affected microtubule structure and found that in comparison with the CTL cells, the length of EB3 comets was significantly shorter in the MAP4-KO cells and unchanged after hyperosmotic stimuli (Figure 5, F and G). We then measured whether hyperosmotic stimuli could induce a calcium response in the MAP4-KO cells and found that deletion of MAP4 abolished the hyperosmotic calcium response (Figure 5, H and I). Addition of thapsigargin, an inhibitor of the ER calcium ATPase, increased cytosolic calcium in the MAP4-KO cells, indicating the cells were able to respond to calcium agonists (Supplemental Figure 9D). The phenocopied results between the PC2-KO and MAP4-KO cells demonstrate that deletion of either of these molecules impairs the ability of the cell to sense hyperosmotic stimuli and trigger a calcium response.

### The PC2-mediated osmosensing pathway is required for AQP2 membrane insertion.

Urine concentration, in part, depends on the uptake of water through AQP2 whose trafficking is activated by both AVP and hyperosmolality (44). The expression of AQP2 in patients with ADPKD is increased (45, 46), which is paradoxical to their urinary concentration deficit. Like patients with ADPKD, we observed a significant increase in AQP2 expression in IMCD3 PC2-KO cells that remained unchanged after hyperosmotic stimuli (Supplemental Figure 10, A and B).

**Figure 6:**
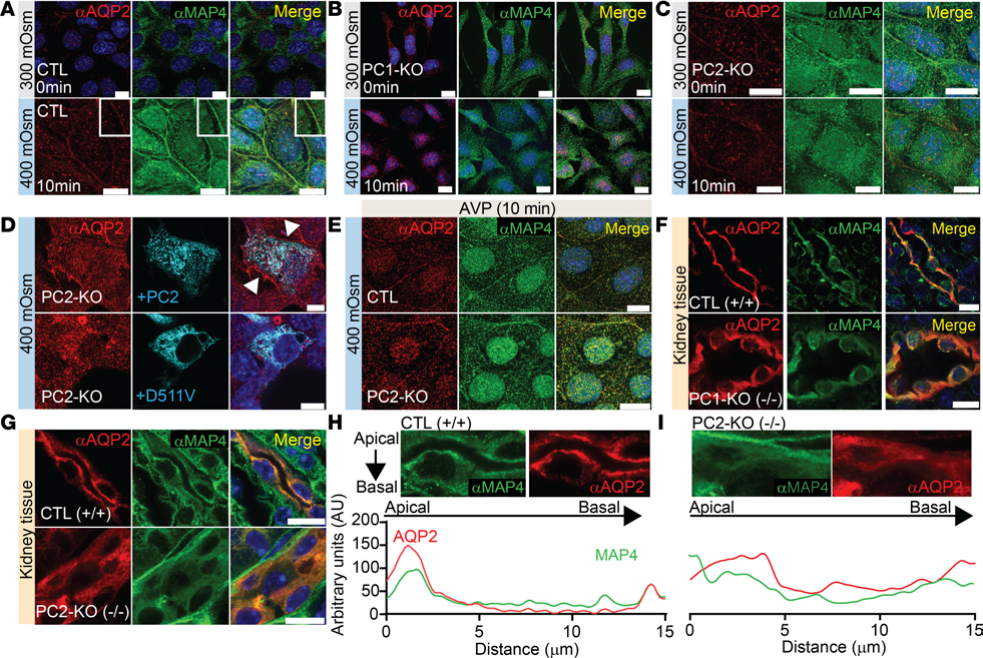
Hyperosmotic aquaporin 2 (AQP2) trafficking to the membrane is impaired in PC1-KO and PC2-KO IMCD3 cells and localizes to the cytoplasm of CDs from PC1-KO and PC2-KO mice. (**A**–**C**) Representative immunofluorescent staining of IMCD3 CTL cells (**A**), PC1-KO cells (**B**), and PC2-KO cells (**C**) labeling AQP2 (red) and MAP4 (green) at 300 mOsm (top) and after hyperosmotic stimuli (400 mOsm, bottom). Scale bars: 10 μm. Hyperosmotic stimuli induced trafficking of AQP2 in the membrane of IMCD3 CTL cells but not in PC1-KO or PC2-KO cells. (**D**) Reexpression of full-length PC2 (cyan, top panel) restored AQP2 (red) trafficking to the membrane after hyperosmotic stimuli but not the PC2-D511V variant (cyan, lower panel) in IMCD3 PC2-KO cells. Scale bars: 10 μm. White arrows highlight AQP2 staining in membrane. (**E**) AQP2 is localized to the membrane with 100 nM AVP in CTL and PC2-KO IMCD3 cells. Scale bars: 10 μm. (**F** and **G**) AQP2 (red) and MAP4 (green) in kidney slices from CTL mice (top panel) and PC1-KO mice (**F**) or PC2-KO mice (**G**) (bottom panels). Scale bars: 10 μm. (**H** and **I**) Line scan of localized apical staining of AQP2 (red) and MAP4 (green) in kidney slices from CTL mice (**H**) versus cytosolic in PC2-KO (**I**) mice.

We then examined AQP2 distribution after hyperosmotic challenge. At baseline in CTL IMCD3 cells, AQP2 predominantly resided in the cytosol, and moved to the plasma membrane after hyperosmotic stimuli, colocalizing with MAP4 (Figure 6A). In contrast, there was minimal distribution of AQP2 and MAP4 in the membrane of PC1-KO or PC2-KO cells after hyperosmolarity (Figure 6, B and C). Reexpression of full-length PC2 but not D511V restored localization of AQP2 in the plasma membrane (Figure 6D). As a control, we stimulated the cells with AVP, and found that in CTL cells, AQP2 localized in the membrane (Figure 6E). Interestingly, in PC2-KO cells, half of the cells showed labeling of AQP2 in the membrane (Figure 6E). We also measured AQP2 cytosolic vesicle size and found that hyperosmotic stimuli significantly increased vesicle size in CTL cells (Supplemental Figure 10C). PC2-KO cells had significantly larger vesicles at baseline, which remained unchanged after hyperosmotic stimuli (Supplemental Figure 9C).

We isolated primary renal CD cells from mice and stimulated both CTL and PC2-KO cells with hyperosmolality. The same phenotype remained after hyperosmotic challenge where AQP2 moved to the plasma membrane of CTL cells but not in PC2-KO cells (Supplemental Figure 10D). Lastly, we confirmed these results by examining the localization of AQP2 on the CD of CTL, precystic PC1-KO and PC2-KO mice. AQP2 and MAP4 in CTL mice colocalized in the membrane (Figure 6, F and G). In comparison, in PC1-KO and PC2-KO mice, there was diffuse cytosolic staining of AQP2 and MAP4 (Figure 6, F–I*)*.

Since MAP4 sits upstream of PC2, we tested whether loss of MAP4 in vivo phenocopied the PC2-KO mice. MAP4 shRNA, under the control of a *cre* promotor, was effective at reducing the MAP4 expression in IMCD3 cells with *cre* as shown by Western blot (Supplemental Figure 11, A and B). After hyperosmotic stimuli, AQP2 predominantly resided in the cytosol of MAP4-KD IMCD3 cells (Supplemental Figure 11C). In kidney sections, MAP4 resided in the CD, as it colocalized with AQP2-positive cells (Supplemental Figure 11D). The *cre*-dependent MAP4 shRNA (and nontargeted control) was packaged into lentivirus and retroorbitally injected into doxycycline-fed Pax8-TetO mice, thus enabling restriction of the shRNA expression to the *cre*-positive kidney tubule. This approach has been previously utilized in kidney tubules (47). The ability of the shRNA to knock down MAP4 in the presence of *cre* was demonstrated via immunofluorescence assay (Supplemental Figure 11E). MAP4-KD mice had a striking decrease in urine concentration 7 days after transduction compared with doxycycline-fed Pax8-TetO mice injected with a lentivirus without a targeted shRNA (Supplemental Figure 11F). More importantly, AQP2 in the CD of the MAP4-KD mice localized to the cytosol, as seen in PC1-KO and PC2-KO mice (Supplemental Figure 11G). Collectively, these results demonstrate the physiological importance of the PC1/MAP4/PC2 osmosensing pathway, as it enables the insertion of AQP2 into the membrane.

**Figure 7:**
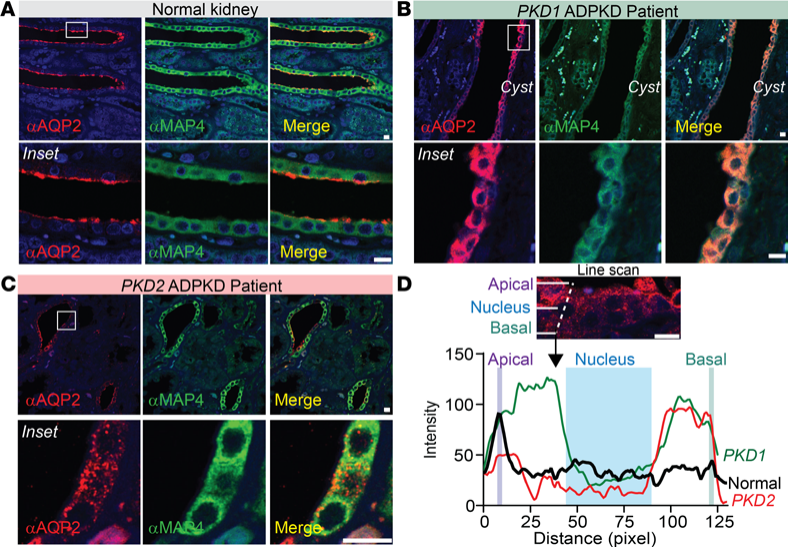
AQP2 is predominantly found in the cytoplasm of CD cells in kidney slices from patients with ADPKD. (**A**–**C**) Immunofluorescent staining of AQP2 (red) and MAP4 (green) (top) and at a higher magnification (inset; bottom panels) in kidney slices from a normal human kidney with AQP2 associated at or near the apical membrane (**A**), but prominent cytosolic labeling in *PKD1* ADPKD patient (**B**) or likely *PKD2* ADPKD patient (**C**). Images represent *n* = 4 patients with ADPKD and 3 normal kidneys. (**D**) Line scan of AQP2 in CD cells with cytosolic and/or basal localization in *PKD1* (green) and *PKD2* (red) ADPKD patients compared with a predominant apical localization in normal kidneys (black). Scale bars: 10 μm (top panels) and 5 μm (bottom panels).

To determine whether this pathway has translational relevance, we examined tissue from 3 normal human kidneys, a patient with a known *PKD2* mutation, and 3 patients who likely have a *PKD1* mutation based on the age of onset of end-stage renal disease (ESRD) (Supplemental Table 3). In the normal kidneys, AQP2 had a prominent localization to the apical membrane of CD cells (Figure 7A). However, in both *PKD2* and *PKD1* ADPKD patients, AQP2 vesicles accumulated within the subapical storage compartment of CD cells, with minimal labeling within the apical membrane (Figure 7, B and C). Regardless of the presence of cysts, AQP2 was primarily localized to the subapical compartment of the cytosol in ADPKD tubules and cyst epithelia (Figure 7D). Lastly, we observed a significant increase in p-MAP4 after hyperosmotic stimuli in cells isolated from normal kidneys (Supplemental Figure 12A). In contrast, ADPKD patient cells had increased p-MAP4 levels under isosmotic conditions that remained unchanged after hyperosmotic stimuli (Supplemental Figure 12A), replicating our in vitro findings. Altogether, these data suggest that loss of polycystin-mediated osmosensing disrupts the trafficking of AQP2 in patients with ADPKD, providing a potential explanation for their urine concentration deficiency.

## Discussion

Our study provides evidence that both PC1 and PC2 mediate a hyperosmotically dependent calcium signal that aids in the trafficking of AQP2 in the CDs. Mechanistically, we show that a mild hyperosmotic stimulus leads to ER-localized PC2 calcium release through a mechanotransduction pathway involving the PC2-MAP4 and microtubule interaction. We found that hyperosmolarity increases the phosphorylation of MAP4, which induces microtubule depolymerization, reduces the PC2-MAP4 interaction, and triggers ER-localized PC2-mediated calcium release. This pathway enables AQP2 membrane insertion in CD cells independently of AVP, thus increasing water reabsorption and concentrating the urine. However, in ADPKD, the functional loss of the osmotically dependent PC1-MAP4-PC2 interaction reduced AQP2 apical membrane insertion and water reabsorption by the CD, leading to a concentrating defect — one of the earliest symptoms in patients with ADPKD.

Despite 30 years of research, the physiological stimuli activating polycystin proteins remain elusive, a key challenge as there is no cure for ADPKD. PC1 and PC2 have been described as mechanosensory proteins in the primary cilia (48), bending in response to changes in fluid flow or sheer stress, releasing a factor or signaling molecules like calcium (49, 50). However, what has not been tested is whether osmolality stimulates the polycystin complex in kidneys. We found that ciliary calcium was not required for the hyperosmotic response. Instead, this hyperosmotic response required ER-localized PC2 to release calcium. This highlights distinct roles for ciliary and ER-localized PC2, with ciliary PC2 sensing fluid flow (49, 50), while ER-localized PC2 is required for osmotically induced calcium release. Thus, our proposed model aligns with the ciliary hypothesis, as it appears that the 2 localizations of PC2 — cilia and ER — can discriminate between the extracellular cues the cells are experiencing. Nonetheless, we cannot rule out that ciliary PC1 and PC2 could be involved in the osmotic activation of signaling pathways that are calcium independent, like Wnt, Notch, and Hedgehog signaling (51) or cilia-dependent cyst activator (CDCA) (52) that then may integrate with the ER-PC2 calcium signaling. Lastly, other investigators have found that in yeast, osmotic stress can activate *Pkd2* and regulate cellular events such as cytokinesis, suggesting a conserved function (53).

The current study provides a mechanistic model in which the polycystin proteins function as an osmotic sensor, triggering distinct temporal steps like cytoskeletal restructuring and calcium release from the ER to modulate water permeability of the CD cells. Hyperosmolarity can induce microtubule reorganization (order of seconds to minutes) (54). The speed of the response seen in our experiments is likely driven by the polycystin-dependent activation of an unknown kinase that facilitates microtubule rearrangement, eliciting a PC2-dependent calcium response, whose channel activity is regulated by cytoskeletal components (55–58). We propose this rearrangement occurs through the phosphorylation of MAP4, as one protein potentially within a complex of microtubular proteins that interact to regulate the PC2-mediated calcium response. This response is supported by evidence showing that deletion of the polycystin proteins affects the phosphorylation status of MAP4. However, we cannot rule out that other phosphorylation sites on MAP4 are involved, or that a complex of additional microtubular proteins interact with ER-localized PC2. Although MAP4 is expressed in muscle cells (36), we show that MAP4 within the kidney is most highly expressed in the AQP2-positive segments; thus, the polycystin-MAP4 interaction is likely to have the most effect in the CD. A limitation of the current study is that the MAP4-KD analysis was confined to urine concentration measurements after 1 week. It remains to be determined what is the chronic effect of MAP4-phosphorylation inhibition on renal function.

Patients with ADPKD experience decreased urinary concentrating ability as an early symptom (8). Paradoxically, previous work has pointed to higher expression of AQP2 in patients with PKD and murine models (45, 46, 59–61). However, these studies did not examine the subcellular localization of AQP2 in the CDs. We found that the PC2-mediated hyperosmotic pathway enabled AQP2 trafficking to the membrane distinct from AVP. As the transport machinery that allows these vesicles to travel along the microtubules are calcium dependent (62), the absence of calcium signals in both PC1-KO and PC2-KO likely impairs the transport machinery of AQP2 trafficking. Indeed, with 3 different mouse models, we show that disruption to any of the proteins in this osmosensing pathway (PC1, MAP4, or PC2) impairs AQP2 trafficking and likely leads to dilute urine compared with their respective controls.

A long-standing unanswered question is why patients with ADPKD have increased circulating AVP levels (5, 6, 63). In cystic biopsies from patients with ADPKD, we found that AQP2 has a prominent subapical cytosolic localization. More importantly, noncystic tubules also displayed increased cytosolic location of AQP2, indicating that this is not a phenomenon of the cystic phenotype. We posit that impaired hyperosmotic AQP2 trafficking impedes water reabsorption in patients with ADPKD, resulting in increased activation of the central AVP pathway in the early stages of the ADPKD. A limitation of our study is the absence of AVP measurements from the patient samples analyzed. However, sustained increased levels of circulating AVP, a known cyst activator via the V2R and cAMP pathways, further promotes cystic pathways (64, 65). Concurrently, tubular damage and cyst growth can then exacerbate disruptions to the medullary gradient in the later stages of ADPKD (8, 66, 67), exacerbating the concentrating defect phenotype. Many tubular kidney diseases are characterized by impaired urine concentrating deficiencies, with mechanisms such as reduced tubular response to AVP and potential disruption of the osmotic gradient being proposed (68, 69). The role of the polycystin proteins in the context of these other renal disorders has yet to be determined.

In conclusion, our findings provide insights into a physiological role for the polycystin proteins in osmosensing and ER-mediated calcium release to promote AQP2 trafficking. These 2 findings likely explain the impaired urine concentrating ability and elevated circulating AVP levels in patients with ADPKD. Therapeutic strategies that target the PC2-MAP4 regulation of ER-mediated calcium release may prevent early defects in the urine concentrating ability and support lower circulating levels of AVP, to prevent the sustained upregulation of cAMP that promotes cystogenesis and disease progression (70, 71).

## Methods

### Sex as a biological variable.

Our study examined male and female animals, and similar findings are reported for both sexes.

### Animal models.

*Pkd2^fl/fl^* mice (20–22) (gift from Stefan Somlo, Yale University, New Haven, Connecticut, USA) were crossed with TetO-Cre and Pax8-rtTA mice (The Jackson Laboratory, strains 006234 and 007176) to generate Pax8-TetO-rtTA *Pkd2^fl/fl^* mice and Pax8-TetO-rtTA control mice. Some mice were further crossed with LSL-Salsa6f mice (The Jackson Laboratory, strain 031968) to generate mice that expressed gCaMP6F and tdTomato upon *cre* induction (25). Precystic male and female mice, 13–16 weeks of age (1.5–2 months after induction with 625 mg/kg doxycline chow [Envigo]) were used in the subsequent experiments.

Tissue collection from *Pkd1*-KO mice was conducted at the University of Maryland. *Pkd1^fl/fl^* were crossed with TetO-Cre and Pax8-rtTA mice to generate *cre*-positive and -negative Pax8-TetO-rtTA *Pkd1^fl/fl^*. After genotyping to confirm the expected alleles, *cre* expression was induced by administering 3 intraperitoneal injections of doxycycline (50 mg/kg body weight) on P21, P22, and P23. Urine samples were collected on P35 (14 days after induction). Following euthanasia, kidneys were perfused with saline, fixed for 2 hours using 2% paraformaldehyde (PFA), and embedded in OCT for further histological analysis.

### Urine osmolality analysis and metabolic cage analysis.

Animals provided with ad libitum water were moved to a hydrophobic surface and urine collected in the morning within the first 3 minutes of the animal being removed from the cage. Urine osmolality was measured with a vapor osmometer (Wescor 5520 Vapor Pressure Osmometer). A subset of animals had body measurements conducted by NMR spectroscopy followed by metabolic cage analysis (TSE Systems) to measure energy expenditure, food, and water intake over 5 days.

### Imaging of kidney sections.

Animals were sedated with isoflurane and perfused with saline via the left ventricle. The kidneys were rapidly excised and dissected into approximately 1-mm coronal sections in ice-cold PBS and then transferred to kidney tubule solution (in mM: 120 NaCl, 3 KCl, 2 CaCl_2_, 2 KH_2_PO_4_, 5 glucose, 10 HEPES; pH 7.3). Kidney sections from gCaMP6-expressing mice were imaged after kidney excision, while sections from gCaMP-negative mice were incubated in Fluo4-AM (5 μM; Invitrogen) for 30 minutes at room temperature. Sections were washed 2 times with fresh kidney tubule solution. Medullary tubules were identified by visual inspection based on anatomical landmarks. Images were acquired by incubating the tubules at 250 mOsm (5 minutes) followed by 400 mOsm stimuli (10 minutes). To ensure distal and CD tubules were being assessed, those that responded to exogenously applied AVP were included in the analysis. Ionomycin was added at the completion of the experiment.

### Isolation of murine primary culture CD cells.

Seven-week-old male mice were anesthetized with isoflurane (2%–3%) and perfused with 0.9% saline via the left ventricle. Kidneys were excised and decapsulated under sterile conditions. The medullary regions were dissected and minced with a razor blade and subjected to digestion by collagenase (LS5475, Worthington, ~300 U/mL) and hyaluronidase (LK3240, Worthington ~1,500 U/mL) in minimum essential Eagle’s medium (MEM) with Earle’s salts and 1% penicillin/streptomycin for 45–60 minutes at 37°C with gentle rotation. At the end of the digestion, 1 mL of Hank’s balanced salt solution (HBSS) containing 0.1% BSA and 2 mM EDTA and 1 mL of MEM were added. The cell suspension was triturated gently approximately 5 times and filtered through a 70-μm cell strainer (Falcon). The resultant mixture was spun at 500*g*. The cell pellet was resuspended in MEM with Earle’s salts and 1% penicillin/streptomycin. To purify CD cells, the cells were incubated with gentle rotation for 20 minutes at 4°C with Dynabeads Biotin Binder (11047, Invitrogen) prelabeled with approximately 1 μg of *Dolichos biflorus* agglutinin (DBA), biotinylated (B-1035-5, Vector Laboratories). Beads were trapped on magnets and washed in isolation buffer (Dulbecco’s balanced salt solution, Ca^2+^ and Mg^2+^ free with 0.1% BSA and 2 mM EDTA). The cell pellet was resuspended in Dulbecco’s modified Eagle’s medium (DMEM)/F12 (Cytiva) with 10% fetal bovine essence (FBE) (Avantor Sciences) and 1% penicillin/streptomycin and cultured on coverslips. Media were changed daily. Cells were transduced with lentivirus containing either the nontemplate guide control or *Pkd2* guide sequence with Cas9 as described below. Forty-eight hours after transduction, cells were incubated with Fluo4-AM (4 μM) for calcium imaging experiments or incubated in different osmolality solutions and fixed for immunofluorescence experiments. Analysis was done from 3 different mice and statistical analysis determined as described in *Statistics* below.

### Cell culture and maintenance.

Murine C2C12 myoblasts (used to dissect the molecular mechanism) and HEK293T cells were purchased from ATCC. HEK293 cells that contain all the InsP_3_R isoforms knocked out (known as 3KO-HEK cells) were a gift from David Yule (University of Rochester, Rochester, New York, USA) (36). IMCD3 cells were a gift from Indra Chandrasekar (Sanford Health, University of South Dakota, Vermillion, South Dakota, USA). C2C12 and HEK293T cells were cultured in DMEM (Cytiva), while IMCD3 cells were cultured in DMEM/F-12 media (Cytiva). All media were supplemented with 10% FBE and antibiotics at 37°C and incubated in a humidified atmosphere with 5% CO_2_ and 95% air. Cell cultures were kept to passages no higher than 12.

### Generation of PC1, PC2, and MAP-4 CRISPR KO cell lines.

We tested 2 different CRISPR/Cas9 knockout All-In-One ZsGreen pClip lentivirus plasmids, each containing a separate guide sequence directed at different *Pkd1*, *Pkd*2, and *MAP4* loci, along with a control template (Transomic Technologies). The lentiviruses were made by cotransfecting pRSV, pMDLg, and pMD2.G along with pClip into HEK293T cells. The supernatant containing virus particles was harvested and used to transduce C2C12, IMCD3, or HEK293T cells. Following 48 hours of transduction, ZsGreen fluorescent C2C12, IMCD3, or HEK293T cells were sorted by flow cytometry and single-cell clones expanded. Selection of MAP4 from the mixed population was obtained by supplementing the media with 10 μM blasticidin (Thermo Fisher Scientific). Following expansion, the different cell lines were validated by Western blot, qPCR, and immunofluorescence assays.

### MAP4 lentiviral shRNA constructs and animal models.

Three different shRNAs against MAP4 were selected using the Genetic Perturbation Platform (Broad Institute; https://portals.broadinstitute.org/gpp/public/). A scrambled sequence or the specific MAP4 shRNAs were cloned into the pSICO plasmid (Addgene) that enables shRNA expression under *cre* promoter control. Effectiveness of the shRNA was tested by cotransfecting either the scrambled sequence or the MAP4-shRNA-pSICO plasmid with *cre* into IMCD3 cells and validated via Western blot. We confirmed the expression of both *cre* and the shRNA through the expression of their respective fluorescent proteins. Two of the 3 shRNA sequences were effective, and 1 was selected for lentiviral production. Lentivirus was then produced using a protocol previously described (47). Transduction of the cells was confirmed by GFP expression and MOI was used to determine titers. MAP4 shRNA virus or empty-vector control (1 × 10^9^ titers/mL each) was injected into Pax8-TetO-rtTA mice via retroorbital injection following doxycycline diet. Urine osmolality was measured before injection and 1 week after injection. Animals were then sacrificed and tissue processed for histological analysis.

### Generation of eGFP-MAP4 constructs.

MAP4 (mouse sequence) cDNA was purchased from Transomic Technology Inc. and cloned into a GFP plasmid backbone using Gibson Assembly (New England Biolabs) following the manufacturer’s instructions and validated by PCR and sequencing. The phosphorylation mutations were made by using a Q5 site-directed mutagenesis kit (New England Biolabs) following the manufacturer’s instructions. The correct insertion of the mutation was confirmed by sequencing (Integrated DNA Technologies).

### Measurements of intracellular calcium.

We measured calcium changes in the cytosol, ER, plasma membrane, and cilia. Calcium measurements were measured using the following plasmids: gCAMP6F (cytosol) (27), R-CEPIA (ER; gift from Aleskey Zima, Loyola University Chicago) (33), CAAX-gCaMP7s (plasma membrane; gift from Jordan Beach, Loyola University Chicago) and Arl13B-gCaMP6F (cilia; gift from Aldebaran Hofer, Harvard University, West Roxbury, Massachusetts, USA; and made by Yubin Zhou’s lab) (32). Cells were plated on glass coverslips 48 hours prior to imaging. Cells were transiently transfected 24 hours after plating with 2 mg of the DNA of interest and 25 μL of PEI (1 mg/mL stock; Sigma-Aldrich) diluted in Opti-MEM (Thermo Fisher Scientific) and incubated overnight. Regions of interest were drawn on individual cells to quantify calcium measurements in at least 3 independent biological replicates. Cells were perfused with a gravity exchange fluid system (Warner Instruments) for 2 minutes at 3 mL/min with 300 mOsm solution (in mM: 130 NaCl, 2 CaCl_2_, 1 MgCl_2_, 2 K_2_PO_4_, 5 glucose; 10 HEPES, 0.1 EGTA; pH 7.3) prior to imaging. Cells were then perfused with the specified osmotic solution. Increase in osmotic concentration was done by increasing the concentration of NaCl or mannitol as described in Results. The osmolality of solutions was measured by a vapor pressure osmometer (Wescor 5520 Vapor Pressure Osmometer). gCaMP6F was excited with a 488 nM LED (Lumencor Spectra X Lamp) and emitted fluorescence filtered with a band pass filter (515–530 nm, Chroma). Images were acquired with a sCMOS camera (Orca Flash, Hamamatsu) on a Zeiss wide-field fluorescence microscope. Images were acquired at 15-ms intervals for a total of 3–6 minutes at room temperature.

### Western blot analysis.

Total protein extracts were collected after incubating cells under the different osmotic conditions after 1 hour at 37°C and prepared by lysing cells with RIPA buffer (in mM: 10 Tris-Cl, 1 EDTA, 0.5 EGTA, 1% Triton X-100, 0.1% sodium deoxycholate, 0.1% SDS, 140 NaCl) containing protease inhibitor cocktail (Sigma-Aldrich), and phosphatase inhibitors NaF and sodium orthovanadate (Alfa Aesar). Reexpression of WT PC2-mCherry or PC2-D511V mCherry was performed by transfecting cells 24 hours prior to protein extraction. PC2-D511V was generated by Q5 site-directed mutagenesis (New England Biolabs), following the manufacturer’s protocol. Protein concentrations of the resulting supernatants were measured using the Pierce BCA Protein Assay Kit (Thermo Fisher Scientific). Equal amounts of protein (15–20 mg) were separated by SDS-PAGE (Bio-Rad, 4%–20% gradient gels) and transferred to PVDF membranes via wet transfer. Membranes were probed overnight with the following primary antibodies: α-tubulin (1:1,000; 2125S; Cell Signaling Technology), GAPDH (1:1,500; 6004-Ig; ProteinTech), PC2 (1:500; clone D-3, sc-28331, Santa Cruz Biotechnology) (26, 72–74), MAP4 (1:1,000; 11229-1-AP, ProteinTech) (75–78), p-MAP4 (1:1,000; Ser696, A51201, https://www.antibodies.com/), GFP, CLIMP63 (1:1,200; clone CKAP4, A302-257A, Fortis Life Science), AQP2 (1:1,000; 3487, Cell Signaling Technology). HRP-conjugated secondary antibodies were applied (Immun-Star goat anti-mouse, 1705047, 1:20,000; and Immun-Star goat anti-rabbit, 1705046, 1:20,000, both Bio-Rad) and then activated with Clarity Max Western ECL (Bio-Rad). Chemiluminescence was imaged with a ChemiDoc MP imager (Bio-Rad); signal intensity of each protein was measured with Image Lab software (Bio-Rad, v.6.0) and normalized to total protein or a loading control (Ponceau S). At least 3–5 biological replicates were analyzed.

### Immunofluorescence microscopy.

Cells were grown on coverslips and cultured in media for 24 hours. Cells subjected to osmotic stimulus were incubated in the specified osmotic buffer for 5–10 minutes and immediately fixed. Cells were fixed in 2% PFA for 20 minutes at room temperature, washed 3 times in PBS, and blocked for 45 minutes in 2% BSA blocking solution with 0.2% Triton X-100. The cells were incubated with antibodies against PC2-YCE2 (1:100; sc-47734, Santa Cruz Biotechnology), MAP4 (1:100; sc-390286, Santa Cruz Biotechnology), CLIMP63 (1:2,000; clone CKAP4, A302-257A, Fortis Life Science), AQP2 (1:1,000; 3487, Cell Signaling Technology), and Arl13b (1:100; 17711-1-AP, ProteinTech) overnight at 4°C, followed by the appropriate secondary antibody, Alexa Fluor 488 donkey anti-mouse IgG (1:1,000; A21202, Invitrogen) or Alexa Fluor 546 donkey anti-rabbit IgG (1:800; A10040, Invitrogen), for 1 hour, and then washed 3 times in PBS. Some slides were coincubated with Phalloidin-iFluor 647 Conjugate (1:1,000; 20555, Cayman Chemical) during the secondary incubation step. Coverslips were mounted with ProLong Diamond mounting medium with DAPI (Invitrogen). After curing, cells were imaged with a 43× oil (N.A. 1.2) or 63× oil (N.A. 1.4) objective on an LSM 880 laser-scanning microscope with Airyscan (Zeiss). Images were postprocessed with Zen Black software (Zeiss) and FIJI (NIH) (79).

### Human tissues.

ADPKD and normal human kidney tissues were obtained by the PKD Biospecimen and Biomaterials Core in the Kansas PKD Center at the University of Kansas Medical Center (KUMC) and the PKD Research Resource Consortium (PDK-RRC). Tissues were fixed with 4% PFA at 4°C overnight, embedded in paraffin, and 5-μm sections were cut. Following deparaffinization and rehydration, antigen retrieval was performed by incubating the sections in heated sodium citrate buffer (10 mM tri-sodium citrate, 0.05% Tween 20, pH 6.0). Sections were then quenched of autofluorescence, blocked, permeabilized, and incubated with anti-MAP4 (Santa Cruz Biotechnology and ProteinTech) and anti-AQP2 (Cell Signaling Technology) antibodies. Tissues were mounted in ProLong Diamond Antifade Mountant (Life Technologies), and counterstained with DBA. Due to the lack of available DNA, genotyping was not performed. The classification of the samples was based on clinical and pathological criteria. Due to the relatively young age at onset of ESRD in this cohort, it is more likely that these patients carry mutations in *PKD1* rather than *PKD2*, since *PKD1* mutations are typically associated with more severe disease and earlier progression to kidney failure, whereas *PKD2*-associated disease tends to present later, with an average age of ESRD around 70 years (80–82). It is also unlikely other genes are involved, as minor genes are typically associated with a milder disease (83, 84).

### Live-cell imaging of microtubules and plus-end microtubules.

Cells were plated on glass coverslip 48 hours prior to imaging. Tracking of the plus-end side of the microtubules was performed by transiently transfecting the cells with EB3-tdTomato (Addgene, 50708) 24 hours after plating, following the protocol described in *Measurements of intracellular calcium* above. Cells were imaged with a 63× oil objective in an LSM 880 Zeiss laser-scanning microscope with Airyscan.

### Analysis of live-cell imaging.

Calcium imaging movies were analyzed using FIJI and the Time Series Analyzer V3 plugin. Quantification of the velocity, duration, and length of EB3-tdTomato comets was performed using the MtrackJ plugin in FIJI (85).

### Immunoprecipitation and MS.

Protein (100 μg) isolated from the left ventricle and kidney of WT C57BL/6 mice was incubated with PC2 antibody (10 μg; Alomone Laboratories) complexed to magnetic protein G beads (Bio-Rad) overnight. Following 3 rounds of washing, the bound protein was eluted and run alongside flow-through in 4%–12% polyacrylamide gels. Silver stain (Bio-Rad) was used to identify bands for excision. Gel bands were excised and desiccated under high vacuum. Gel bands (corresponding to 20, 30, 40, 70, 110, 200, and 250 kDa) were forwarded for LC-MS/MS analysis. This analysis was carried out using an LTQ Orbitrap Elite coupled with UltiMate 3000 RSLCnano system at Rosalind Franklin University (facility run by Charlie Yang). In-gel tryptic digestion was followed by the identification of proteins with the UniProt mouse database and data processed by PEAKS 8.5 software (Bioinformatics solutions Inc.). Data were filtered based on –10^logP^, FDR, unique peptide, and de novo ALC%. As an internal control, 6 bovine standard proteins (β-lactoglobulin, lactoperoxidase, carbonic anhydrase, glutamate dehydrogenase, α-casein, and serum albumin) were identified with high scores under the same conditions, to ensure that LC-MS/MS was running properly. Immunoprecipitation of MAP4 and PC2 was performed by incubating 100 μg of protein lysate with either PC2 antibody (1 μg; Santa Cruz Biotechnology) or MAP4 antibody (1 μg; ProteinTech) preincubated with magnetic beads (IgG, Bio-Rad). For eGFP, Chromotrek GFP-trap magnetic agarose beads (ProteinTech) were used following the manufacturer’s instructions.

### Statistics.

Data were plotted using GraphPad Prism 9. Where *n* values of a group were less than 15, nonparametric distribution was assumed. Where the *n* values of a group exceeded 15, data were tested for normality using a normality and distribution test. For parametrically distributed data comparing 2 independent groups, 1-tailed Student’s *t* test was used. For nonparametric data, Mann-Whitney *U* test was conducted. Comparison of multiple groups with parametric distribution were analyzed by 1-way ANOVA followed Šídák’s multiple-comparison test. Comparison of multiple groups with nonparametric distribution were analyzed by Kruskal-Wallis test followed by Dunn’s test. Conditions were considered statistically significant when *P* values were less than 0.05. For live-cell imaging experiments, quantifications were conducted using 10–20 cells per experimental condition from 3–5 biological replicates. For immunofluorescence, analysis was performed on 2–3 biological replicates and at least 10 cells were used to determine the average. Error bars indicate SEM.

### Study approval.

All animal studies performed were done under the Institutional Animal Care and Use Committee–approved protocols at Loyola University Chicago and University of Maryland. The use of ADPKD and normal human kidney tissues was approved by the IRB at KUMC (not considered to be human subjects research by regulatory agencies) and under exemption at Loyola University Chicago.

### Data availability.

Values for all data points in graphs are available and presented in the Supporting Data Values file.

## Author contributions

KMMN and IYK conceived the study. KMMN, RMK, VV, CY, PO, DPW, and IYK designed and performed the experiments. KMMN and IYK performed the analysis. KMMN, RMK, VV, DPW, and IYK participated in the discussion of the manuscript. KMMN and IYK wrote and revised the manuscript. All authors approved the final manuscript.

## Supplementary Material

Supplemental data

Unedited blot and gel images

Supplemental video 1

Supplemental video 2

Supplemental video 3

Supporting data values

## Version 1 08/05/2025

In-Press Preview

## Version 2 09/23/2025

Electronic publication
