# Supplementary material for: Hyperosmotic stimuli activate polycystin proteins to aid in urine concentration
Source: JCI Insight. 2025 Aug 5;10(18):e186290. doi: 10.1172/jci.insight.186290 (PMC12487848; doi:10.1172/jci.insight.186290)
Supplement: Supplemental data [file jciinsight-10-186290-s134.pdf]

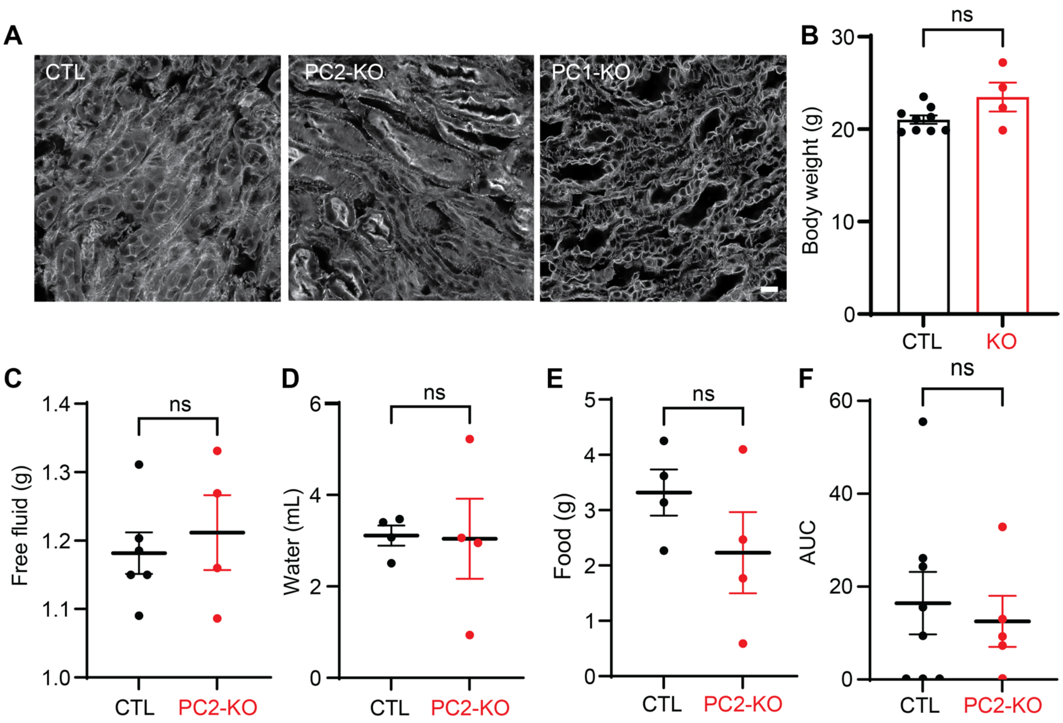

Supplemental Figure 1. Physiological properties in CTL and tubule specific PC2-KO mice. A. Representative images of kidneys from CTL, PC2-KO and PC1-KO mice staining with phalloidin show no cysts. Scale bars represent 10μm. **B.** Body weight of CTL and PC2-KO mice in metabolic cages. **C.** No change in bodily free fluid of CTL and PC2-KO mice in metabolic cages. **D-E.** No change in water (**D**) or food (**E**) consumption during 24h of CTL and PC2-KO mice in metabolic cages. **F.** No changes in AUC of cytosolic calcium increase in CTL and PC2-KO CD tubules after vasopressin addition. Bar graphs represent mean±SEM. Dots represent individual mice with an n=4-9. Statistical analysis was determined by Mann-Whitney U test. p-values are listed in each panel.

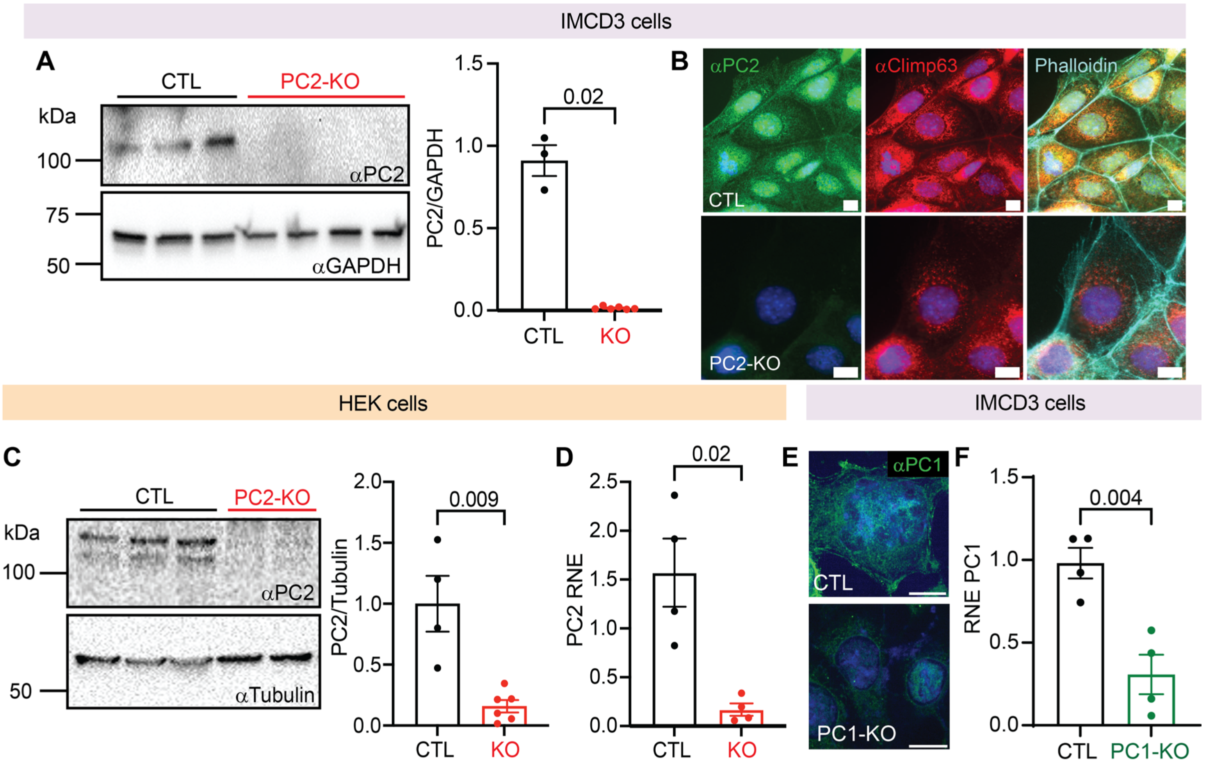

Supplemental Figure 2. Validation of PC2 and PC1 knockout in renal cell lines. A. Representation immunoblot and analysis showing PC2 expression was significantly decreased in IMCD3 PC2-KO cells in comparison to CTL cells. GAPDH was used as loading control. **B.** Co-localization of PC2 (*green*) and CLIMP63 (*ER morphology; red*) confirms its localization to the ER in IMCD3 CTL (*top panel*). PC2 expression is absent in PC2-KO cells (*bottom panels*). Phalloidin staining is presented in cyan. Scale bars represent 10μm. **C.** PC2 expression was significantly decreased in PC2-KO HEK cells compared to the CTL cells. Tubulin was used as loading control. **D.** mRNA of PC2 was significantly decreased in PC2-KO HEK cells. **E.** Immunofluorescent assay of CTL and PC1-KO IMCD3 cells. PC1 expression is absent in PC1-KO cells (*bottom panels*). **F.** mRNA of PC1 was significantly decreased in PC1-KO IMCD3 cells. Bar graphs represent mean±SEM. Statistical analysis was determined by Mann-Whitney U test. p-values listed in the panels. Dots represent biological replicates with n=3-4.

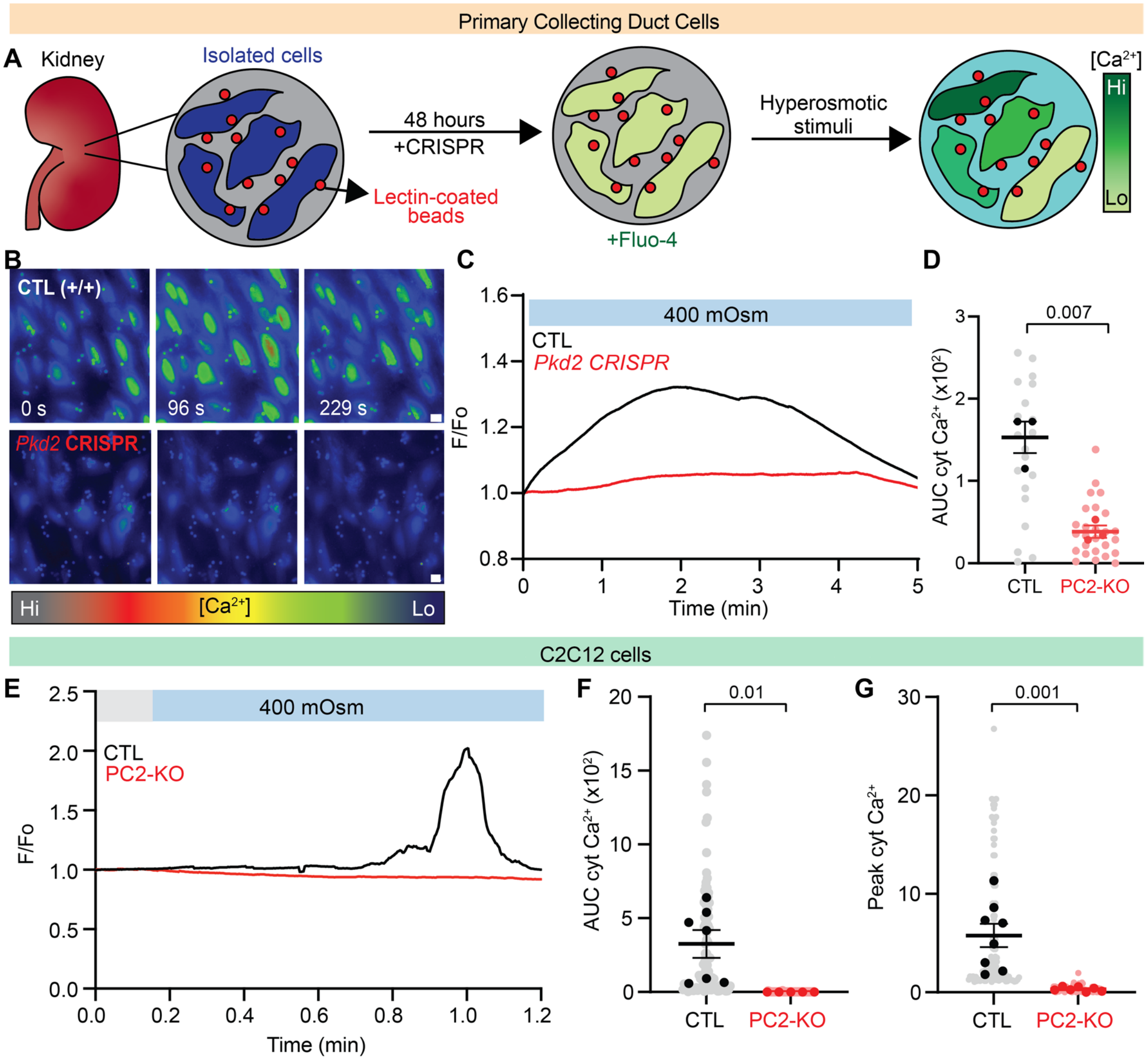

Supplemental Figure 3. Loss of PC2 abolished cytosolic calcium response to hyperosmotic stimuli. A. Diagram of isolation of primary renal CD cells. **B.** Representative time lapse images of CTL primary renal CD cells (*top panels*) and *Pkd2*-CRISPR-KO (*bottom panels*) after hyperosmotic stimuli. Scale bars represent 10μm. **C.** Representative trace of cytosolic calcium increase in CTL primary renal CD cells (*black line*) but not in the *Pkd2*-CRISPR-KO CD cells (*red line*) from panel *B.* **D.** Quantification of AUC was significantly decreased in *Pkd2*-CRISPR-KO primary renal CD cells in comparison to the CTL cells. **E.** Representative trace of cytosolic calcium changes in CTL

(*black line*) and PC2-KO C2C12 cells (*red line*). Cytosolic calcium increase was absent in PC2-KO cells. **F.** AUC was significantly decreased in C2C12 PC2-KO cells. **G.** Peak cytosolic calcium was significantly decreased in the C2C12 PC2-KO cells. Bar graphs represent mean±SEM. Dots represent individual cells from at least three independent biological replicates. Data was first analyzed to determine normality then followed with an unpaired t-test. p-values are listed in the panels. n=3-8 biological replicates. Dark dots represent biological replicates while grayed dots represent individual cells.

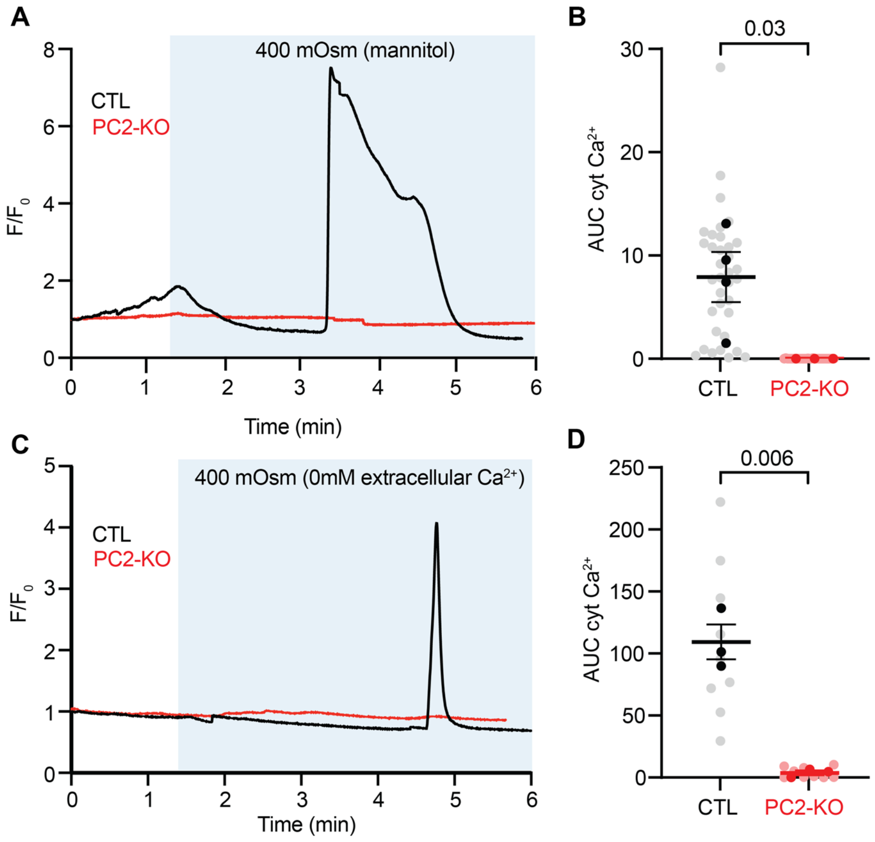

Supplemental Figure 4. Hyperosmotic stimuli induced intracellular calcium release. **A.** Representative trace of cytosolic calcium changes in C2C12 CTL (*black line*) and PC2-KO cells (*red line*) after increase of extracellular osmotic concentration using mannitol. Cytosolic calcium increase was absent in PC2-KO cells. **B.** AUC was significantly decreased in C2C12 PC2-KO cells. **C.** Representative trace of cytosolic calcium increases in C2C12 CTL cells (*black lines*) which are absent in PC2-KO cells (*red line*) after hyperosmotic stimuli with 0mM extracellular calcium. **D.** AUC was significantly decreased in C2C12 PC2-KO cells. Bars represent mean±SEM. Data were analyzed to determine normality. Statistical analysis was determined by an unpaired t-test. p-values in figure. n=3-4 biological replicates. Dark dots represent biological replicates while grayed dots represent individual cells.

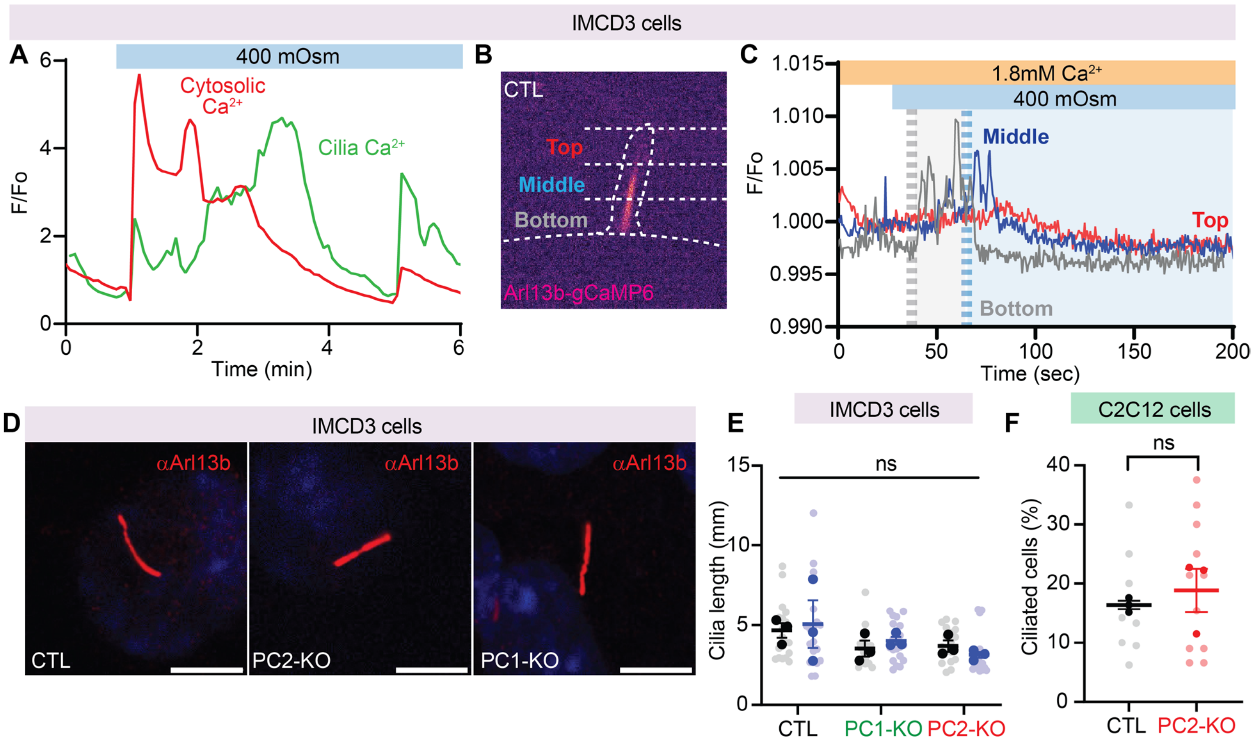

Supplemental Figure 5. Hyperosmotic stimulus does not stimulate ciliary calcium signals. A. Representative trace of cytosolic (*red line*) and ciliary calcium (*green line*) after hyperosmotic stimuli. **B.** Representative image of Arl13b-gCaMP6F and section analyzed (*top, middle and bottom*). **C.** Representative traces of the three regions outlined in *Panel B*. Increase of ciliary calcium first appeared in the bottom section of the cilia followed by the middle section indicative of backpropagation. **D.** Representative immunofluorescent staining of Arl13b in CTL, PC2 and PC1-KO IMCD3 cells. **E.** No significant difference was observed in ciliary length of CTL, PC1 and PC2-KO IMCD3 cells. **F.** Quantification of the percentage of cilia per total number of cells was similar between C2C12 CTL and PC2-KO cells. Bar graphs represent mean±SEM. Statistical analysis was determined by Mann-Whitney U test. p-values listed in panels. n=3-4

biological replicates. Dark dots represent biological replicates while grayed dots represent individual cells.

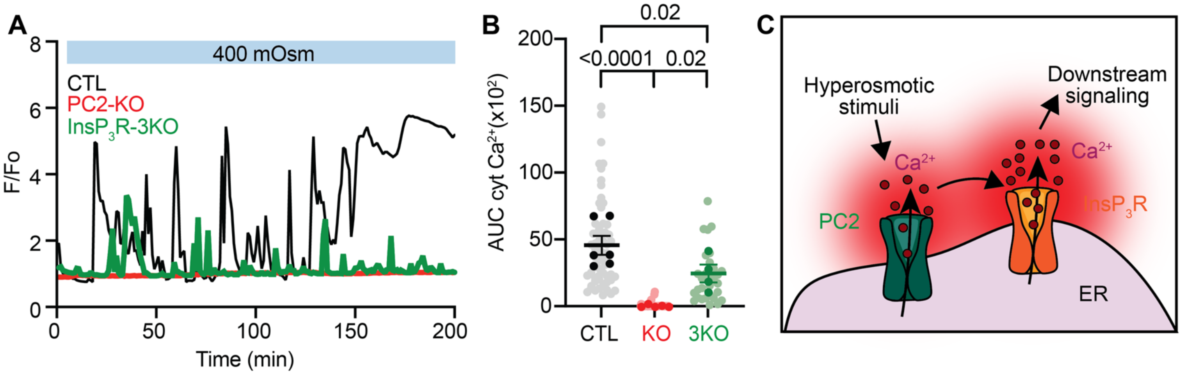

Supplemental Figure 6. PC2 potentially recruits InsP3R to sustain the hyperosmotically induced cytosolic calcium response. A. Representative trace of cytosolic calcium changes in CTL (*black line*), PC2-KO (*red line*) and 3KO-HEK (*green line*) cells. Cytosolic calcium increase was absent in PC2-KO cells. **B.** AUC was significantly decreased in both PC2-KO and 3KO-HEK cells. Bars represent mean±SEM. Statistical analysis was determined by One-way ANOVA followed by Kruskal-Wallis analysis (Gaussian distribution was not assumed); p-values listed. **C.** Hyperosmotic stimuli require an initial calcium release mediated by PC2 which is further sustained by recruiting InsP3R localized in the ER. n=30-40 cell from 4-6 biological replicates. Dark dots represent biological replicates while grayed dots represent individual cells.

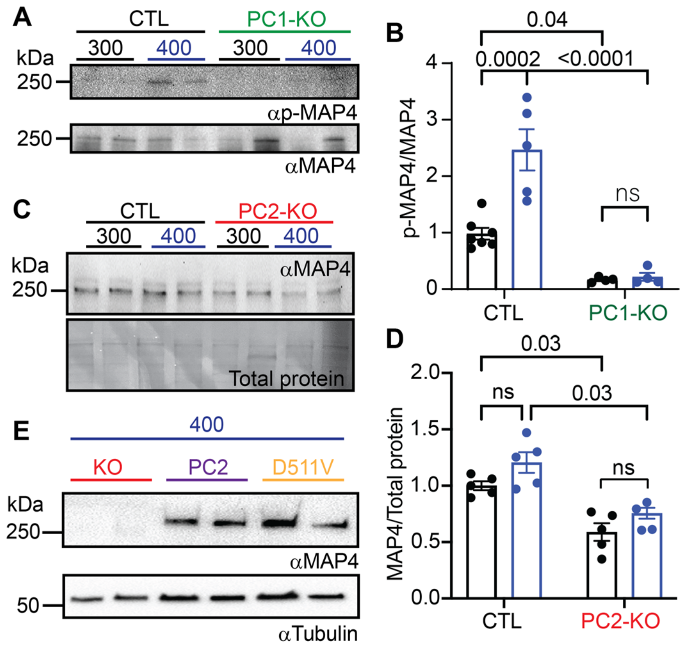

Supplemental Figure 7. Phosphorylation of MAP4 is polycystin dependent. A. Western blot of p-MAP4 and total MAP4 expression in CTL and PC1-KO imCD3 cells. **B.** p-MAP4/MAP4 ratio was significantly decreased in PC1-KO imCD3 cells in comparison to CTL cells. Statistical analysis was determined by Kruskal-Wallis test followed by Dunn's test. **C.** Western blot of total MAP4 expression in C2C12 CTL and PC2-KO cells. Total protein was used as loading control. **D.** Total MAP4 expression is significantly decreased in C2C12 PC2-KO cells in comparison to the CTL cells. Statistical analysis was determined by Kruskal-Wallis test followed by Dunn's test. **E.** Expression of MAP4 in C2C12 PC2-KO cells (*red*), PC2-KO + PC2 (*PC2, purple*) and PC2-KO + PC2-D511V (*D511V; orange*) at 400 mOsm. Statistical analysis was determined by Kruskal-Wallis test followed by Dunn's test. GAPDH was used as loading control. n=4-5 biological replicates.

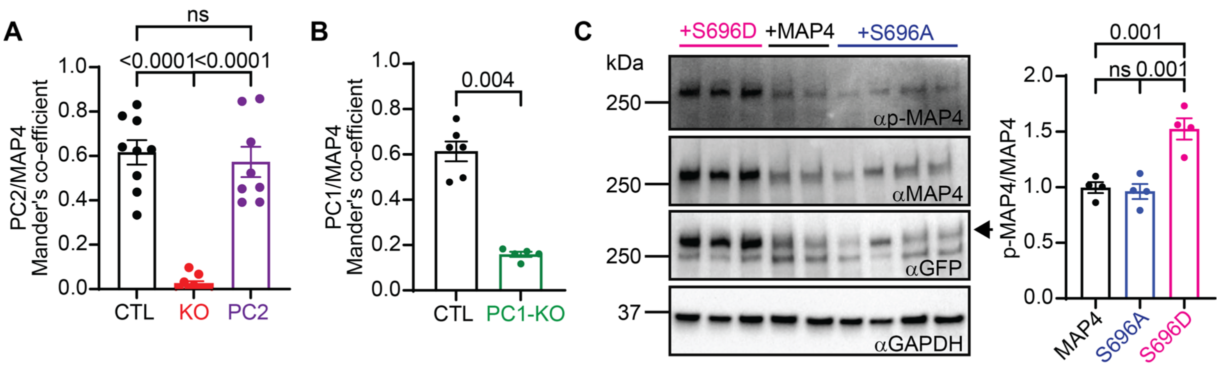

Supplemental Figure 8. Localization and interaction of MAP4 and PC2. A. Quantification of Mander's co-efficient of PC2/MAP4 in C2C12 CTL and PC2-KO cells. **B.** Quantification of Mander's co-efficient of PC1/MAP4 in IMCD3 CTL and PC1-KO cells. Bar graphs represent mean±SEM with listed p-values. Images shown on Figure 3. **C.** Western blot showing expression of p-MAP4 and its phospho-mutants. GAPDH was used as loading control. p-MAP4/MAP4 ratio is significantly increased in imCD3 PC2-KO cells over-expressing the phospho-mimetic variant S696D but not the S696A phospho-null variant. Statistical analysis was determined by Kruskal-Wallis test. n=4-9 biological replicates.

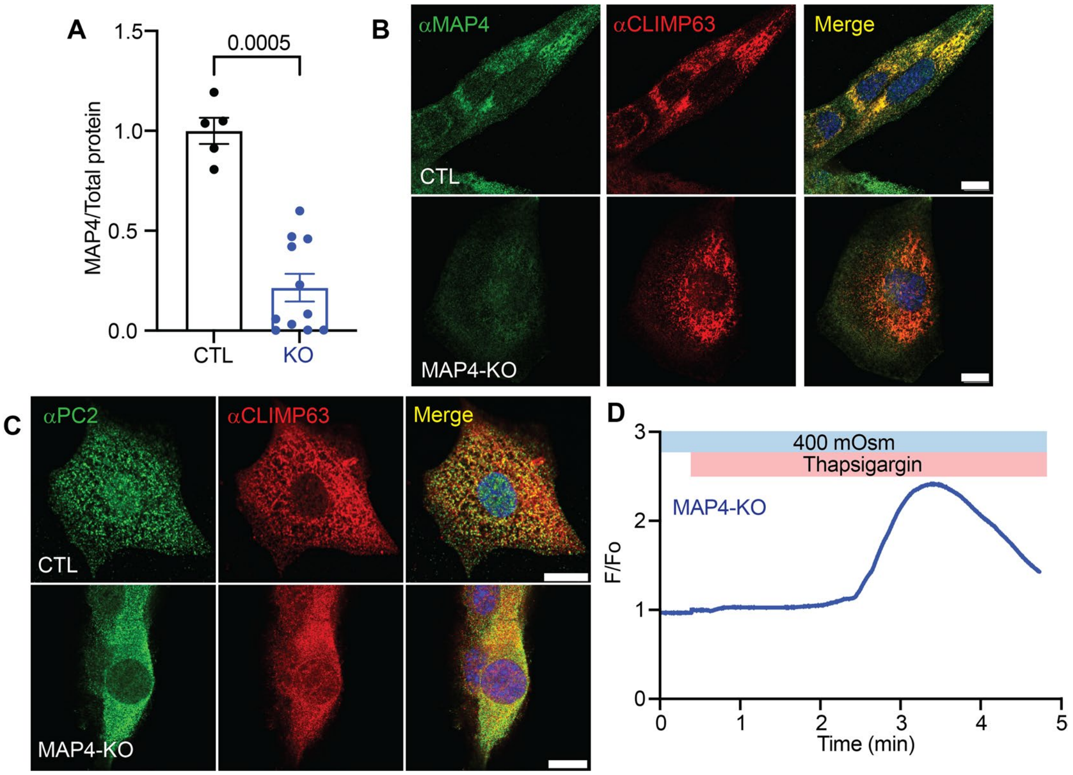

Supplemental Figure 9. MAP4-KO phenocopies osmosensitive response of PC2-KO cells. A. MAP4 expression decreased 75% in CRISPR-Cas9 knockout cell line. Bars represent mean±SEM. Data were analyzed to determine normality. Statistical analysis was determined by Mann-Whitney U test. n=5-11 biological replicates, p-values listed. **B.** Immunofluorescence staining of MAP4 and CLIMP63 in C2C12 CTL cells (*top panels*) and MAP4-KO cells (*bottom panels*). Scale bars represent 10μm. **C.** Immunofluorescence staining of PC2 and CLIMP63 in C2C12 CTL cells (*top panels*) and MAP4-KO cells (*bottom panels*). **D.** Representative trace of cytosolic calcium increase using thapsigargin after hyperosmotic stimuli in MAP4-KO cells.

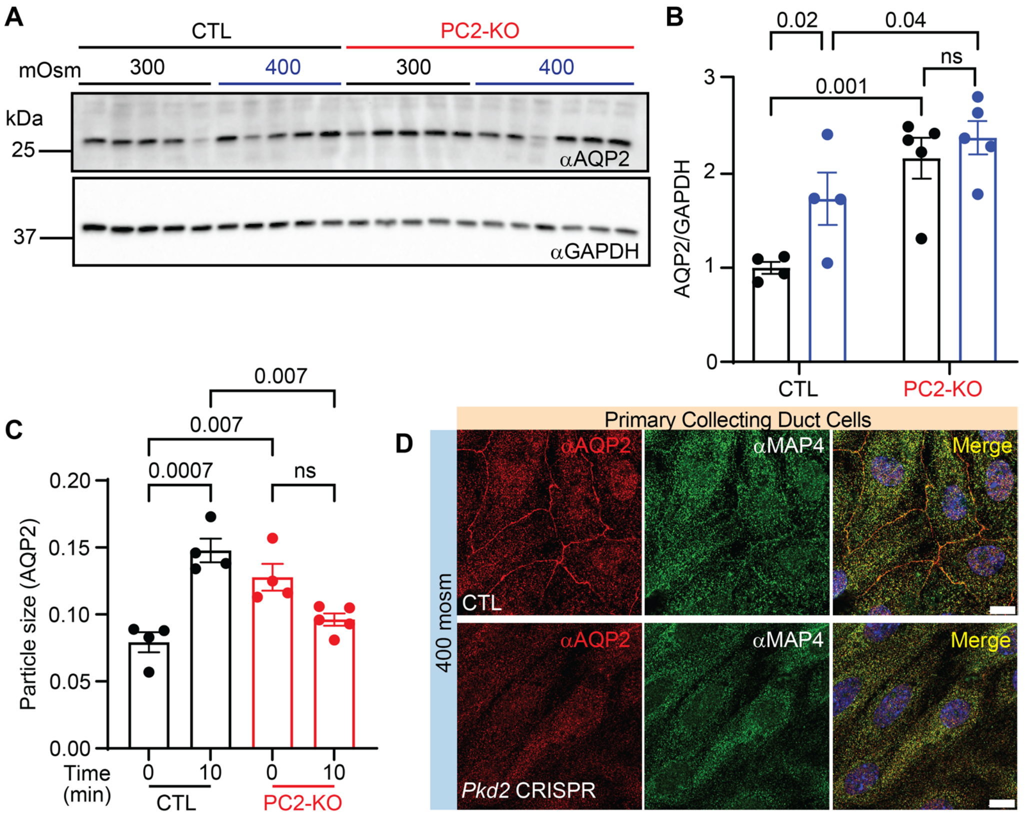

Supplemental Figure 10. AQP2 expression is increased in PC2-KO cells. A. Expression of AQP2 at baseline (300mOsm) and after hyperosmotic stimuli (400mOsm) in IMCD3 CTL and PC2-KO cells. GAPDH was used as loading control. **B.** Quantification of AQP2 expression. Statistical analysis was determined by Kruskal-Wallis test followed by Dunn's test. **C.** AQP2 vesicle size significantly increases after hyperosmotic stimuli in IMCD3 CTL cells but not in PC2-KO cells. Statistical analysis was determined by Kruskal-Wallis test followed by Dunn's test. **D.** Immunofluorescent staining of primary renal CD cells of AQP2 (*red*) and MAP4 (*green*) after hyperosmotic stimuli in CTL (*top panels*) and *Pkd2* CRISPR-KO cells (*bottom panels*). Scale bars represent 10μm. n=4-5 biological replicates.

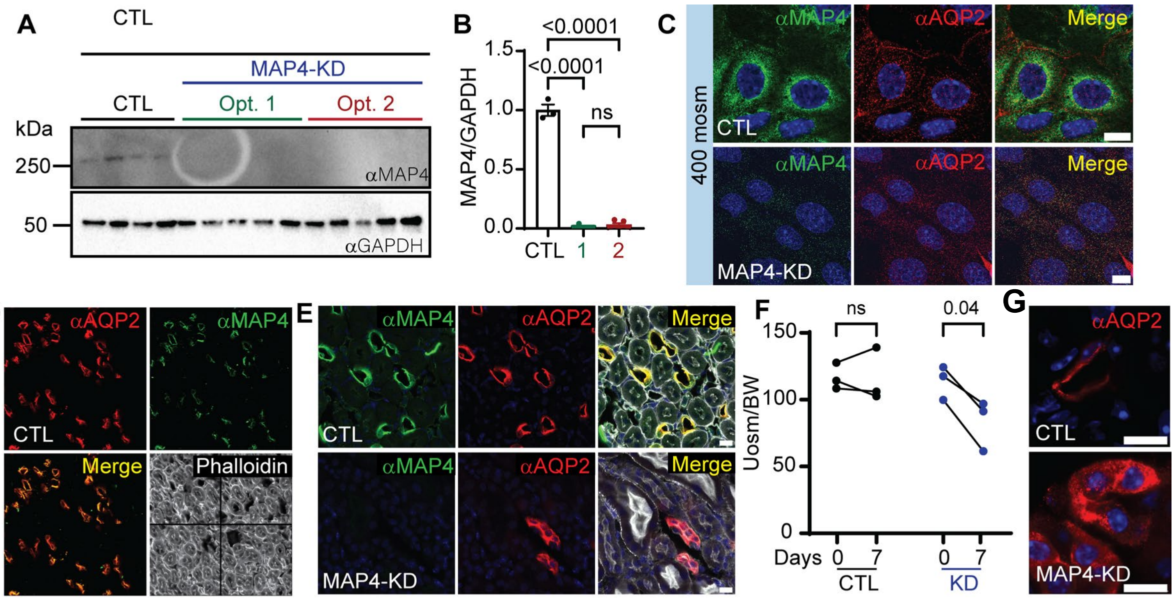

Supplemental Figure 11. MAP4 knockdown disrupts AQP2 trafficking and results in dilute urine. A. Western blot of total MAP4 after transduction of lentivirus containing Cre-inducible shRNA of MAP4-KD in IMCD3 cells transfected with a Cre-construct. GAPDH was used as loading control. **B.** MAP4 expression was significantly decreased in MAP4-KD IMCD3 cells transduced with two options (Opt.) of MAP4-shRNA virus. Statistical analysis was determined by Kruskal-Wallis analysis. n=4-5 biological replicates. **C.** Immunofluorescent staining of MAP4 (green) and AQP2 in IMCD3 CTL (*top panels*) and Map4-shRNA lentivirus transduced cells (*bottom panels*). **D.** Immunofluorescent staining of AQP2 (*red*) and MAP4 (*green*) in kidney slices from CTL mice. **E.** Immunofluorescent staining of MAP4 (*green*) and AQP2 (red) in kidney slices from CTL mice and MAP4-KD mice. Scale bars represent 10μm. **F.** MAP4-KD mice had decreased urine osmolality in comparison to the CTL mice. Each dot represents an individual mouse; n=3. Data analyzed by Mann-Whitney U test. **G.** AQP2 (*red*) in kidney slices from CTL mice and MAP4-KD mice. Scale bars represent 10μm.

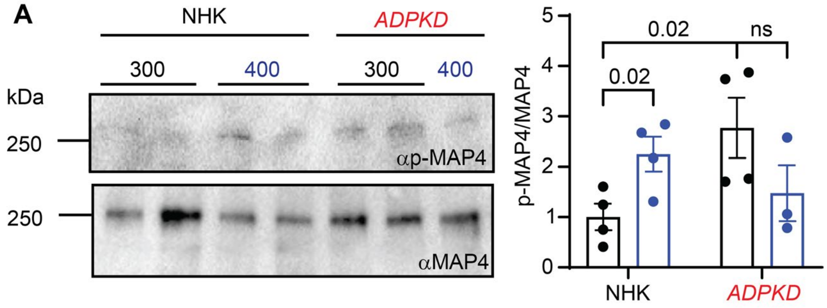

Supplemental Figure 12. p-MAP4 expression is increased in cells isolated from ADPKD kidneys. A. Expression of p-MAP4/MAP4 ratio was significantly increased after hyperosmotic stimuli in normal human kidneys (NHK) cells but unchanged in ADPKD patient cells. n=4 biological replicates. Bars represent mean±SEM with p-values.

Supplemental Table 1. Protein list of PC2 immunoprecipitation mass spectrometry analysis.

Accession	Coverage (%)	Peptides	Avg. Mass	Description
**Q62261|SPTB2_MOUSE**	19	39	274221	Spectrin β chain non-erythrocytic 1
**P15508|SPTB1_MOUSE**	15	25	245248	Spectrin β chain erythrocytic
**A2AQP0|MYH7B_MOUSE**	6	34	221495	Myosin-7B
**P16546|SPTN1_MOUSE**	11	22	284596	Spectrin α chain non-erythrocytic 1
**P70670|NACAM_MOUSE**	13	19	220497	Nascent polypeptide-associated complex subunit alpha muscle-specific form
**P27546|MAP4_MOUSE**	25	15	117429	Microtubule-associated protein 4
**P02468|LAMC1_MOUSE**	9	11	177298	Laminin subunit gamma 1
**O55143|AT2A2_MOUSE**	7	6	114858	Sarcoplasmic/endoplasmic reticulum calcium ATPase 2

Supplemental Table 2. EB3 kinetics in C2C12 CTL and PC2-KO cells.

Parameters	Cell lines	300 mOsm	400 mOsm
**Average length (μm)**	CTL	3.08 ± 0.65	1.13 ± 0.32 ****
	PC2-KO	1.40 ± 0.24 ****	1.18 ± 0.16
**Average velocity (μm/s)**	CTL	0.18 ± 0.06	0.35 ± 0.10 ****
	PC2-KO	0.56 ± 0.06 ****	0.40 ± 0.06 ****
**Duration of comet (s)**	CTL	32.26 ± 9.88	9.49 ± 4.52 ****
	PC2-KO	9.28 ± 2.52 ****	10.20 ± 3.42

Mean ± SEM, **** p <0.0001. Statistical analysis performed by Krustal-Willis followed by Dunn's test.

Supplemental Table 3. Characteristics of ADPKD patients analyzed.

Genotype	Age	Sex	BUN	Creatinine	eGFR	AQP2 Score[A]
PKD2	51	Male	30	3.48	19	3
**Likely *PKD1***	52	Male	40	4	Unknown	2
**Likely *PKD1***	54	Male	42	4.95	12	3
**Likely *PKD1***	55	Male	83	16.35	3	3
**Normal**	56	Male	Unknown	2.3	Unknown	1
**Normal**	53	Male	14	1.3	58	1
**Normal**	53	Male	9	1,01	Unknown	1

A: *AQP2 Score: 1 (Completely apical); 2 (Apical and cytosolic); 3 (Mostly cytosolic).*

Supplementary Video 1: Osmotic calcium induced rise in collecting ducts from renal tubules of CTL and PC2-KO mice.

Supplementary Video 2: Osmotic calcium induced rise in CTL and PC2-KO immortalized murine collecting duct cells (IMCD3).

Supplementary Video 3: Movement of microtubules with osmotic stimuli in CTL and PC2-KO cells.
