# Supplementary figures and images for: Hyperosmotic stimuli activate polycystin proteins to aid in urine concentration
Source: JCI Insight. 2025 Aug 5;10(18):e186290. doi: 10.1172/jci.insight.186290 (PMC12487848; doi:10.1172/jci.insight.186290)

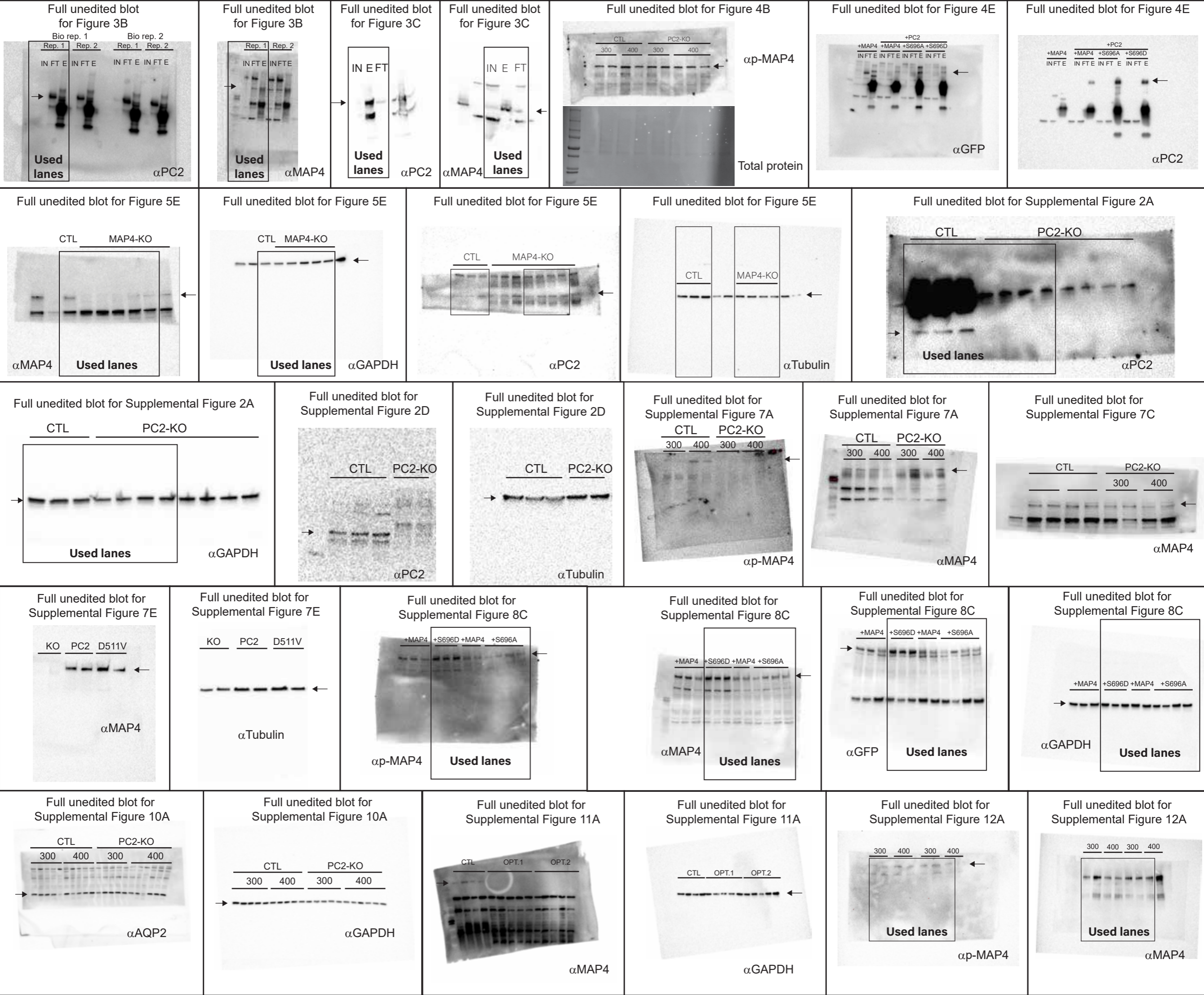

Supplement: Unedited blot and gel images [file jciinsight-10-186290-s135.pdf]
